# Anisotropic Microgels by Supramolecular Assembly and Precipitation Polymerization of Pyrazole‐Modified Monomers

**DOI:** 10.1002/advs.202204853

**Published:** 2022-10-30

**Authors:** Frédéric Grabowski, Vladislav S. Petrovskii, Fabian Fink, Dan Eugen Demco, Sonja Herres‐Pawlis, Igor I. Potemkin, Andrij Pich

**Affiliations:** ^1^ Institute of Technical and Macromolecular Chemistry RWTH Aachen University 52074 Aachen Germany; ^2^ DWI – Leibniz Institute for Interactive Materials 52074 Aachen Germany; ^3^ Institute for Inorganic Chemistry RWTH Aachen University 52074 Aachen Germany

**Keywords:** anisotropic microgels, precipitation polymerization, pyrazole‐modified monomers, self‐assembly, supramolecular interactions

## Abstract

Soft colloidal macromolecular structures with programmable chemical functionalities, size, and shape are important building blocks for the fabrication of catalyst systems and adaptive biomaterials for tissue engineering. However, the development of the easy upscalable and template‐free synthesis methods to obtain such colloids lack in understanding of molecular interactions that occur in the formation mechanisms of polymer colloids. Herein, a computer simulation‐driven experimental synthesis approach based on the supramolecular self‐assembly followed by polymerization of tailored pyrazole‐modified monomers is developed. Simulations for a series of pyrazole‐modified monomers with different numbers of pyrazole groups, different length and polarity of spacers between pyrazole groups and the polymerizable group are first performed. Based on simulations, monomers able to undergo *π*–*π* stacking and guide the formation of supramolecular bonds between polymer segments are synthesized and these are used in precipitation polymerization to synthesize anisotropic microgels. This study demonstrates that microgel morphologies can be tuned from spherical, raspberry‐like to dumbbell‐like by the increase of the pyrazole‐modified monomer loading, which is concentrated at periphery of growing microgels. Combining experimental and simulation results, this work provides a quantitative and predictive approach for guiding microgel design that can be further extended to a diversity of colloidal systems and soft materials with superior properties.

## Graphical abstract

Computer simulations predict supramolecular formation of pyrazole‐modified monomers. Based on the simulations, selected monomers are used in the template‐free and facile upscalable precipitation polymerization to obtain anisotropic microgels (from spherical, raspberry‐like to dumbbell‐like). The results show that the combination of intermolecular *π*–*π* interactions, hydrophobicity, concentration, spacer length, and addition mode of monomer influences the morphology variation.

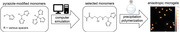

## 1 Introduction

Colloidal nano‐ and microgels are most versatile colloidal building blocks for the design of stimuli‐responsive soft materials,^[^ ^1^ ^]^ which have important applications in catalysis,^[^ ^2^, ^3^, ^4^ ^]^ delivery of active molecules,^[^ ^5^, ^6^, ^7^ ^]^ photonics,^[^ ^8^ ^]^ and biomaterials.^[^ ^9^, ^10^ ^]^ Their versatility stems from the crosslinked, 3D polymer network structure, which is swollen in a solvent^[^ ^11^, ^12^, ^13^ ^]^ (typically water) that can maintain colloidal stability.^[^ ^1^, ^14^, ^15^ ^]^ Microgels are highly biocompatible^[^ ^16^, ^17^ ^]^ and those based on poly(*N*‐isopropylacrylamide) (PNIPAm) or poly(*N*‐vinylcaprolactam) (PVCL) exhibit a volume phase transition temperature (VPTT) around 32 °C in water.^[^ ^12^, ^15^ ^]^ By modification of the microgel structure with specific molecular switches, they may respond to other external stimuli such as pH,^[^ ^18^, ^19^ ^]^ light,^[^ ^20^, ^21^ ^]^ or magnetic field.^[^ ^22^, ^23^ ^]^

Efficient control over the chemical composition, number of crosslinks and size for spherical microgels can be realized using different upscalable synthesis methods like precipitation polymerization, miniemulsion polymerization or emulsion polymerization.^[^ ^24^, ^25^, ^26^ ^]^ Recently, a broad range of complex internal morphologies (core–shell, hollow, etc.) for spherical microgels have been reported.^[^ ^27^, ^28^, ^29^ ^]^

In contrast, the synthesis of microgels with nonspherical shapes is limited to low‐throughput template‐based synthesis approaches as microfluidics^[^ ^30^ ^]^ or lithography.^[^ ^31^ ^]^ Microfluidic processes allowed fabricating rod‐ and snowman‐shaped microgels driven by the shape of the microfluidic channel, the flow velocity or a two‐step polymerization.^[^ ^32^, ^33^ ^]^ Even more complex shapes, such as hook‐ and snowflake‐shaped microgels, were achieved using stop‐flow lithography, whereby the liquid remains stationary during polymerization.^[^ ^31^ ^]^ A major influence on the shapes have the light intensity and exposure time, among other factors.

Anisometric microgels can also be prepared by particle replication in nonwetting templates (PRINT).^[^ ^34^, ^35^ ^]^ Compared to traditional imprint lithography, in PRINT the nonwetting material and surface enclose the liquid precursor in the mold, enabling the generation of isolated particles. The PRINT method allows the fabrication of microgels with high flexibility in size and shape such as conical‐, arrow‐,^[^ ^34^ ^]^ and rod‐shaped^[^ ^35^ ^]^ microgels. However, the method is a time‐consuming multistep process.

The development of the easy upscalable and template‐free synthesis methods to obtain microgels with programmed shapes is hindered by the lack of understanding of interactions at the molecular level in polymerization systems and formation mechanisms of polymer colloids. Nature utilizes programmed molecules and controlled chemical reactions combined with structure formation by supramolecular, hydrophobic, and electrostatic interactions to design complex molecular objects and materials with hierarchical structure, controlled shape, size, and chemical composition.^[^ ^36^, ^37^, ^38^, ^39^, ^40^ ^]^ Lehn et al. effectively used the *π*–*π* stacking interaction capabilities of pyridine–pyridazine oligomers to achieve preorganized helices capable of leading to well‐defined larger polymer architectures upon subsequent polymerization.^[^ ^41^, ^42^ ^]^ Similar synthesis strategies have been used to design various molecular objects,^[^ ^43^, ^44^, ^45^ ^]^ like amphiphilic metallohosts building cigar‐like structures,^[^ ^46^ ^]^ bifunctional ureidotriazines forming helical columns,^[^ ^47^, ^48^ ^]^ and modified heterobicyclic bases organized to helical rosette nanotubes.^[^ ^49^ ^]^ A novel method was developed recently by our group, where a coacervation process was combined with semibatch precipitation polymerization to synthesize Janus‐like polyampholyte microgels.^[^ ^50^ ^]^

In this study, we developed the computer simulation‐driven experimental synthesis approach to obtain anisotropic microgels based on the self‐assembly driven by supramolecular interactions followed by polymerization of tailored pyrazole‐modified monomers. This facile synthesis approach was accomplished by designing a matrix of pyrazole‐modified monomers exhibiting different number of pyrazole groups and various spacers between polymerizable group and pyrazole groups to modulate the accessibility and polarity. First, we used simulations to evaluate the ability of pyrazole‐modified monomers to undergo *π*–*π* stacking. Based on the simulations, we synthesized monomers of pyrazolyl methacrylamide short (PMA‐S), bis(pyrazolyl) methacrylamide short (BPMA‐S), and tris(pyrazolyl) methacrylamide short (TPMA‐S), which differ only in respect of their pyrazole groups, while the spacer length was kept short. In addition, bis(pyrazolyl)‐monomers with longer spacer, bis(pyrazolyl) methacrylate long (BPMA‐L), and higher polarity, bis(pyrazolyl) methacrylate long polar (BPMA‐LP), were synthesized. Afterwards, these monomers were incorporated as comonomers into PVCL‐based microgels with varied amounts (5, 10, and 15 mol%) via batch and semibatch precipitation polymerization. Using advanced microscopy techniques, we demonstrate that the strength of the *π*–*π* interactions between pyrazole groups, the concentration of pyrazole modified monomers in the reaction mixture and the mode of polymerization process (batch vs semibatch) are key parameters that allow programming the shape of microgels. The developed synthesis method allows upscalable synthesis of microgels with programmable shapes using an industrially relevant polymerization technique that enables the design of tailored microgels to fabricate novel drug‐delivery systems, sustainable catalyst carriers, and advanced biomaterials.

## 2 Results

### 2.1 Computer Simulation‐Driven Monomer Synthesis

**Figure 1:**
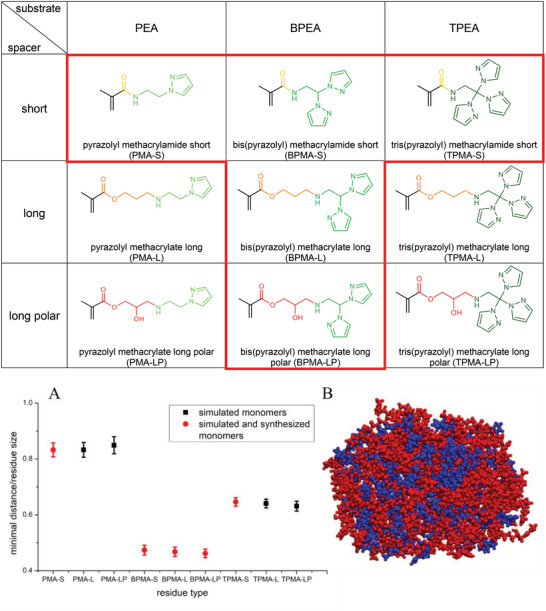
(Table) Pyrazole‐modified monomers considered in this study with variable number of pyrazole groups (green) and different spacers between polymerizable methacrylate group and pyrazole groups: short (yellow), long (orange), or long polar (red). All displayed monomers were used for computer simulations and those outlined in red were synthesized and incorporated in microgels. A) The average minimal distance between pyrazole groups in monomer aggregates as a function of the number of pyrazole groups in monomer structure. B) Snapshot of the equilibrium state of a mixture of VCL (blue) and BPMA‐LP (red) monomers in water/methanol solvent. The solvent molecules are not shown. Such mixtures were used to compute the distance between the pyrazole groups.

For the development of a new microgel synthesis approach involving supramolecular interactions and polymerization, we have decided to use functional monomers decorated with pyrazole groups. Our hypothesis was that the *π*–*π* interactions between pyrazole groups enhance the formation of supramolecular bonds during precipitation polymerization, leading to polymerization‐induced self‐assembly of comonomer‐rich polymer segments and the consequent formation of anisotropic microgels. Organic molecules containing pyrazole groups are well studied in the literature and exhibit strong affinity to form supramolecular structures by *π*–*π* interactions.^[^ ^51^, ^52^, ^53^ ^]^ To systematically investigate the influence of the monomer structure on the morphology of microgels, we considered monomers containing a different number of pyrazole groups (pyrazolylethanamine [PEA], bis(pyrazolyl)ethanamine [BPEA], and tris(pyrazolyl)ethanamine [TPEA]) and variable spacers between pyrazole group and polymerizable group (short (‐S), long (‐L), and long polar (‐LP)) (**Figure** **1** table).

For all these monomers, all‐atom molecular dynamic simulations were performed to evaluate *π*–*π* stacking phenomena and to clarify how the chemical structure and concentration in solution influence the formation of supramolecular structures. The force field we used allows us to take into account *π*–*π* interaction without involving additional quantum calculations. To clarify interactions between the pyrazole monomers (Figure 1 table) we studied their behavior in the presence of *N*‐vinylcaprolactam (VCL) monomers in water/methanol solvent. All monomers were taken in the same proportion (100 VCL monomers:100 pyrazole monomers, Table S20, Supporting Information). In all cases, they formed an almost spherical aggregate after some equilibration time (Figure 1 bottom). We computed the minimal distance between the pyrazole groups in the aggregate to examine the interaction strength and the ability of *π*–*π* pair formation. Since the size of monomers depends on the number of pyrazole groups, the minimal distances were divided by the average size of the monomers (Figure 1A). The minimal possible distances were obtained for the case of bis‐pyrazole. This value is about 0.5 for all spacers, which means that bis‐pyrazole residues placed at the half of the monomer size. This can be explained by the fact that bis‐pyrazole stays planar during the simulation while tris‐pyrazole has a tetrahedral form that interferes with *π*–*π* interactions. Moreover, TPMA monomers assemble into triplets (Figure S15, Supporting Information) and, thus, weaken their *π*–*π* interactions additionally. On the other hand, PMA monomers have only one pyrazole group, which leads to the highest value of the specific minimal distance suggesting weaker *π*–*π* interactions (Figure 1A).

From the simulation results, we selected the monomers PMA‐S, BPMA‐S, TPMA‐S, BPMA‐L, and BPMA‐LP to be synthesized and subsequently incorporated in microgels (Figure 1 top). First, the required substrates PEA and BPEA were synthesized according to literature (Supporting Information).^[^ ^54^, ^55^ ^]^ For the substrate TPEA, the precursor tris(pyrazolyl)methane (TPM) was deprotonated with *n*‐butyllithium and reacted with *N‐*(bromomethyl)phthalimide.^[^ ^56^ ^]^ The resulting product was used to perform a standard hydrazinolysis giving the desired TPEA (Supporting Information). Afterward, the substrates were pre‐modified to ensure a covalent bonding and to achieve an influence of supramolecular interactions during the microgel synthesis. The monomers with short spacers were prepared with methacryloyl chloride (MAC) (Scheme S1, Supporting Information) by a nucleophilic substitution. The BPMA‐L was prepared via a two‐step synthesis, where in the first step an esterification of 3‐bromo‐1‐propanol takes place. In the following step, an amine alkylation with BPEA was performed to obtain the desired BPMA‐L (Scheme S1, Supporting Information). For the final monomer, BPEA was functionalized with glycidyl methacrylate (GMA) by standard nucleophilic addition. During the nucleophilic addition, the primary amine of BPEA was able to react with the *α*‐ or *β*‐carbon atom of the epoxy group of GMA (Scheme S1, Supporting Information). This led to the two corresponding reaction products 3‐((2,2‐di(1*H*‐pyrazol‐1‐yl)ethyl)amino)‐2‐hydroxypropyl methacrylate long polar (*α*‐BPMA‐LP) and 2‐((2,2‐di(1*H*‐pyrazol‐1‐yl)ethyl)amino)‐3‐hydroxypropyl methacrylate long polar (*β*‐BPMA‐LP). Nuclear magnetic resonance (NMR) spectroscopy verified a successful synthesis and revealed a selectivity of 86% for the *α*‐BPMA‐LP. Accordingly, *α*‐BPMA‐LP was obtained as the main product, and a further separation of the *α*‐BPMA‐LP and *β*‐BPMA‐LP was unnecessary. In the following, the new comonomer mixture will be referred to as BPMA‐LP, and only the structure of the main product will be presented.

### 2.2 Microgel Synthesis Guided by Computer Simulations

**Figure 2:**
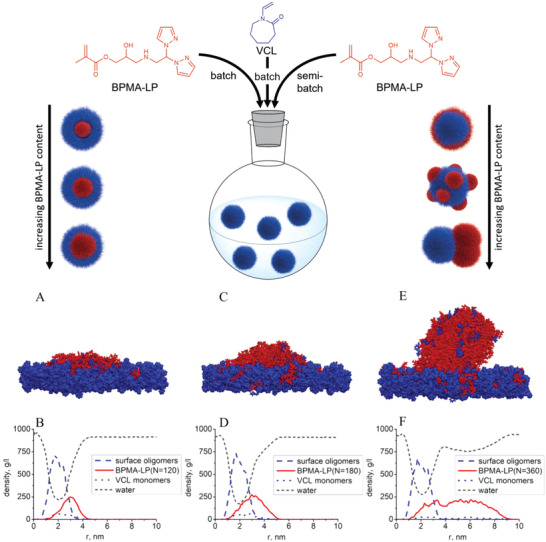
(Top) Schematic synthesis of microgels containing the comonomer BPMA‐LP with different morphologies via batch (B‐MG‐BPMA‐LP, on left) and semi‐batch (SB‐MG‐BPMA‐LP, on right) precipitation polymerization. A,C,E) Snapshots and B,D,F) density profiles of BPMA‐LP monomers at PVCL‐based surface, ordered by increasing content of BPMA‐LP. The BPMA‐LP monomers are shown in red and VCL oligomers (surface) and monomers are in blue. Water and methanol molecules are not shown. Density profiles are calculated in the *z*‐direction (along the normal to the surface). The systems differ by BPMA‐LP concentration (number *N* of the monomers in the simulation box).

Each of the synthesized monomers was subsequently used as comonomer for microgel synthesis along with the main monomer VCL (**Figure** **2** top). We hypothesized that the addition mode of the pyrazole‐modified comonomers may influence the localization of the pyrazole groups in microgels and consequently the microgel morphology can be varied. Using batch precipitation polymerization PVCL microgels with 5, 10, and 15 mol% comonomer content were synthesized. The methacrylamide and methacrylate groups in comonomer structure are more reactive compared to the vinyl group of *N‐*vinylcaprolactam.^[^ ^57^, ^58^ ^]^ The higher hydrophobicity and reactivity of comonomers leads to their faster polymerization and thus to incorporation in the core of the microgels.^[^ ^15^, ^59^ ^]^ Therefore, we expected that core‐shell microgels with comonomer in the core will be synthesized via batch precipitation polymerization (B‐MG‐comonomer, Figure 2 top). To obtain comonomer at the periphery of the microgels, we used semibatch polymerization mode (SB‐MG‐comonomer), where the comonomer was added after polymerization initiation to ensure its localization on the surface of the microgel‐precursor. In this case, due to the higher molecular mobility of pyrazole‐modified monomers induced by smaller number of crosslinks in the microgel‐precursor shell, our expectation was to obtain anisotropic microgels.

These hypotheses were first evaluated by computer simulations. In order to clarify the mechanism of the molecular assembly during the microgel formation, we have considered a planar PVCL‐based surface, which mimics a surface fragment of the microgel‐precursor. The planar geometry is a good approximation, because the size of the microgel‐precursor is much larger than the size of the simulation box. Also, the surface is considered permeable for the BPMA‐LP monomers. The equilibrium distribution of the BPMA‐LP monomers on the PVCL surface is shown in Figure 2 bottom at different values of the monomer concentration (different number of the monomers in the simulation box: *N* = 120, 180, and 360). A detailed description of computer experiments and results for the other monomers are presented in the Supporting Information (Computer Simulations section). Here, we show only the BPMA‐LP based systems (Figure 2 bottom), because they cover the most behavior diversity of all pyrazole monomers. Better affinity of BPMA‐LP monomers toward water than VCL oligomers and monomers (surface) and mixing ability of the different monomers with each other leads to the formation of a thin BPMA‐LP‐rich layer above the PVCL‐rich layer in case of smallest BPMA‐LP concentration (*N* = 120, Figure 2A,B). This concentration value resembles the regime of full spreading of the BPMA‐LP liquid on the VCL surface. Therefore, we conclude that the small BPMA‐LP concentration is responsible for the formation of a homogeneous shell as shown in Figure 2 top. Increase in BPMA‐LP concentration (*N* = 180, Figure 2C,D) leads to the regime of partial wetting. Thus, we expect that replication of the simulated system at larger length scales will result in few droplets laying on the surface. Some of them can coalesce due to the surface tension of the hydrophobic monomers. However, their complete merging into one aggregate is less probable because it requires their motion on the surface at high distance for long enough time. As a result, we expect formation of the raspberry‐like structure upon crosslinking (Figure 2 top). Finally, further increase in BPMA‐LP concentration (*N* = 360, Figure 2E,F) resembles regime of partial wetting of the droplet with small enough contact area. We also predict that replication of the system will lead to more readily coalescence of the droplets due to their bigger volume and ultimately to a dumbbell‐like structure of the microgels upon crosslinking. It should be noted that only the BPMA‐LP system shows the change in the wetting quality with changing in the concentration. All other monomers demonstrated the same wetting regimes at different monomer concentrations (Figures S14 and S15, Supporting Information).

**Figure 3:**
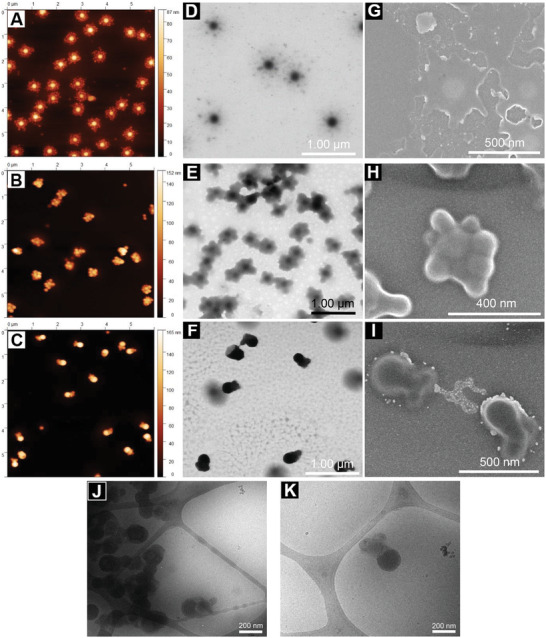
A–C) AFM, D–F) STEM/TEM, and G–I) SEM images of the SB‐MG‐BPMA‐LP microgels ordered by an increase of BPMA‐LP content. At the top are the microgels with 5 mol% (A, D, G), in the middle with 10 mol% (B, E, H) and at the bottom with 15 mol% (C, F, I) of BPMA‐LP. Revealing an increase of the asymmetric shape with increasing BPMA‐LP content from spherical, raspberry‐like to dumbbell‐like shaped microgels. J) Cryo‐TEM images show the agglomeration of the SB‐MG‐BPMA‐LP 15 mol% microgels and K) confirm their anisotropic dumbbell‐like structure.

To confirm the morphological prediction from the computer simulation results, we used various microscopic techniques to evaluate the morphology of synthesized microgels. All experimental results can be found in the Supporting Information. **Figure** **3** presents microscopy images for the SB‐MG‐BPMA‐LP microgels containing different BPMA‐LP amounts at the periphery. The atomic force microscopy (AFM) images show a narrow size distribution of synthesized microgels. It is obvious that the increase of BPMA‐LP content induces strong change of the microgel morphology (Figure 3A–C). The microgels with the lowest BPMA‐LP content have a spherical core surrounded by many small spherical particles (Figure 3A). Similar morphology was observed in our previous work on PVCL‐GMA microgels with 5 mol% GMA content synthesized by semibatch precipitation polymerization.^[^ ^60^ ^]^ However, with further increase of the pyrazole comonomer content, raspberry‐like shaped microgels are obtained (Figure 3B) and the shape for the microgels with 15 mol% BPMA‐LP can be best described as dumbbell‐like (Figure 3C). It should be noted that the respective shapes are formed very selectively, and other shapes are hardly or not visible.

For further investigation, we used scanning transmission electron microscopy (STEM) and transmission electron microscopy (TEM) for the characterization of these microgels, confirming the narrow size distribution for the spherical (Figure 3D), raspberry‐like (Figure 3E), and dumbbell‐like shaped (Figure 3F) microgels. The dark spots in the center of the microgels with 5 and 10 mol% BPMA‐LP indicate region with higher crosslinking density. Consequently, and as a result of the subsequent addition of BPMA‐LP, the microgels consist of a highly crosslinked PVCL core.

In addition, scanning electron microscopy (SEM) of single microgels point out that the microgels with the lowest BPMA‐LP amount have a broad fuzzy outer layer due to the low crosslinking of the corona (Figure 3G). For the raspberry‐like microgels, it is evident that these consist of a big spherical particle coated with many small spherical patches (Figure 3H). The smaller particles vary in size and appear to be well‐separated from the core. In the case of dumbbell‐like shaped microgels (Figure 3I), the main body appears to be composed of a large spherical particle and the tail of smaller particles. In addition to AFM and SEM we used cryogenic TEM (cryo‐TEM) to ensure that different microgel morphologies are not influenced by drying and agglomeration phenomena.

Figure 3J,K shows the cryo‐TEM images of microgels with 15 mol% BPMA‐LP confirming the dumbbell‐like shape. Furthermore, variations in contrast can be observed due to the different crosslinking densities in the microgel. Essentially, the microgels have two domains (Figure 3K). The domain with the higher contrast indicates a higher crosslink density and has a spherical shape. This leads to the assumption that this domain consists of PVCL, since during synthesis VCL can initially only react with the cross‐linker, resulting in a higher crosslink density. The results accordance to the observations made from the SEM images. Overall, the microscopy images confirm the shape variation for the microgels with the increase of BPMA‐LP amount. We also suppose that the larger, spherical compartment of the different microgels, consists mainly of PVCL and the smaller compartments of BPMA‐LP. For the microgels with comonomer localized in the core, only spherical shapes were obtained, and hence the BPMA‐LP amount had no influence on the structure formation (Figure S8, Supporting Information).

AFM and STEM images were also recorded for the B‐MG‐ and SB‐MG‐PMA‐S, BPMA‐S, TPMA‐S, and BPMA‐L microgels synthesized via both batch and semibatch polymerization techniques. The microscopy images revealed that all microgels have a spherical shape regardless of whether the comonomers were incorporated in the core or at the periphery (Figures S1–S3 and S5, Supporting Information). Nevertheless, it was unexpected that no asymmetry occurred for the SB‐MG‐BPMA‐L microgels, considering that the only chemical structure difference to the BPMA‐LP comonomer is the absence of a hydroxyl group. A possible explanation could be the fact that BPMA‐L is hydrophilic compared to the BPMA‐LP, which is hydrophobic.

In addition, the sizes of the SB‐MG‐BPMA‐LP microgels were determined from the STEM/TEM images (Table S19, Supporting Information). Due to the asymmetric shape of the microgels with 15 mol% BPMA‐LP, the length and width were measured. The microgels with the lowest BPMA‐LP content have a diameter of 462.7 ± 56.7 nm and the diameter decreases to 293.8 ± 26.8 nm for the 10 mol% BPMA‐LP microgels. For the dumbbell‐like shaped microgels, a length of 313.4 ± 22.2 nm and a width of 193.3 ± 21.3 nm was determined. The spherical microgels of B‐MG‐BPMA‐LP have a diameter between 235 and 262 nm (Table S19, Supporting Information). In summary, the microgels size depends on the comonomer amount and is larger when the comonomer is located at the periphery.

Likewise, the sizes of the PMA‐S, BPMA‐S, TPMA‐S, and BPMA‐L microgels were determined from the obtained STEM images and given in Tables S4, S8, S12, and S16 (Supporting Information). Also, for all of them, the microgels with comonomer at the periphery are larger than for the respective comonomers and their amounts.

We propose that the *π*–*π* stacking of the BPMA‐LP‐rich polymer segments, caused by the pyrazole groups from the comonomer, leads to self‐assembly of BPMA‐LP and, thus affects the morphology of the microgels. Essential to our hypothesis was the determination of the comonomer content and the evidence of *π*–*π* stacking for all synthesized microgels.

### 2.3 Validation of *π*–*π* Stacking and Comonomer Content Determination

**Figure 4:**
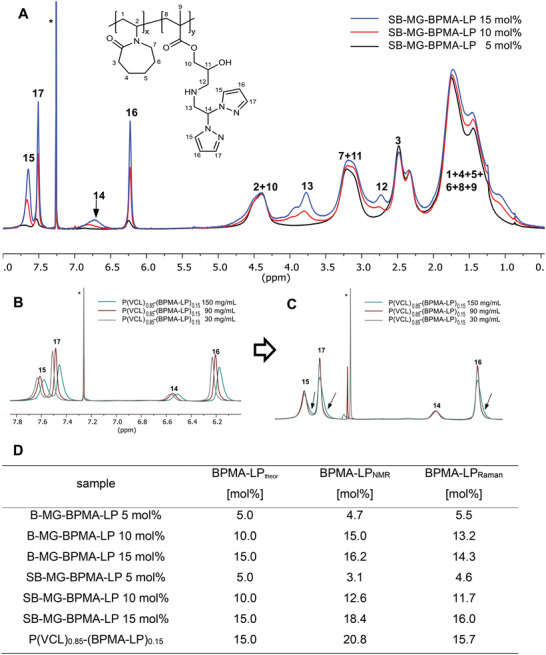
A) ^1^H‐NMR spectra of SB‐MG‐BPMA‐LP microgels measured in CDCl_3_ (*), showing the successful incorporation of the respectively various amounts of BPMA‐LP. B) In the enlarged view of the copolymer P(VCL)_0.85_‐(BPMA‐LP)_0.15_, an increase in the copolymer concentration reveals a shifting of the pyrazole peaks. C) The superimposition shows a broadening for the highest concentration (C, arrows) of the pyrazole groups in the BPMA‐LP structure. D) The table shows theoretical BPMA‐LP content and determined BPMA‐LP content of microgels and copolymer via ^1^H‐NMR and Raman spectroscopy.

We, therefore, used ^1^H‐NMR spectroscopy to analyze the incorporation of the comonomers. The pyrazole peaks of the comonomers have a chemical shift above 6.00 ppm, placing them beyond the PVCL peaks and thus making them clearly visible. For the pyrazole peaks of BPMA‐LP, the intensity correlates with the incorporated BPMA‐LP contents (**Figure** **4**A), confirming the successful incorporation in the microgels. To validate *π*–*π* stacking via NMR, Parenti et al. used different concentrations of homopolymers consisting of thiophene groups leading to a broadening of the corresponding peaks caused by *π*–*π* stacking.^[^ ^61^ ^]^ Furthermore, chemical shifts of the ^1^H‐NMR also indicate *π*–*π* stacking.^[^ ^62^, ^63^ ^]^ The free orientation of polymer chains within microgels is very limited, thus, instead the *π*–*π* stacking of pure copolymer chains was investigated. Therefore, the copolymer P(VCL)_0.85_‐(BPMA‐LP)_0.15_ was synthesized (Supporting Information), and based on the literature different concentrations (30, 90, and 150 mg mL^−1^) were measured via ^1^H‐NMR spectroscopy. The enlarged view with the pyrazole peaks of BPMA‐LP shows a stronger shift with increasing copolymer concentration (Figure 4B). The detection of a possible broadening is hindered due to the shifting of the peaks. In order to observe a broadening, the pyrazole peaks were superimposed revealing a broadening for the copolymer solution with the highest concentration (Figure 4C). The results confirm a *π*–*π* stacking of the pyrazole groups from the comonomer BPMA‐LP and the *π*–*π* interactions enhance with higher concentrations. Also, for the other comonomers, copolymers with 15 mol% comonomer content were prepared and investigated. Similar information was obtained regarding the *π*–*π* stacking. However, for most of the copolymers, a broadening of the pyrazole peaks with increasing copolymer concentration was not observed (Supporting Information). This indicates that *π*–*π* stacking is weaker for the comonomers PMA‐S, BPMA‐S, TPMA‐S, and BPMA‐L than for BPMA‐LP, what is in accordance with the results of computer simulations presented in Figure 1.

Furthermore, the comonomer contents within the microgels and the copolymer were quantified via ^1^H‐NMR spectroscopy. Benzyl benzoate was used as an internal standard for the determination via NMR spectroscopy and the measured contents agree well with the theoretical incorporated amounts of BPMA‐LP in the microgels (Figure 4). The contents for the BPMA‐L microgels are also in good agreement with the theoretical amounts (Table S14, Supporting Information). However, all comonomers with short spacers are significantly above the theoretical amounts. Especially in the case of the batch microgels, with increasing content and number of pyrazole groups of the comonomer, microgels are obtained in which the measured amounts are twice as high and even pure comonomer microgels are obtained for the theoretical highest contents (Tables S2, S6, and S10, Supporting Information). The reason for this is the proximity of the pyrazole groups to the backbone of the polymer chains, whereby the hydrophobicity of the forming polymer chains gets so intense that the VCL can no longer participate in the polymerization. Furthermore, this effect is influenced by the amount of comonomer in the microgel synthesis. Concerning the semibatch microgels with short comonomers, the contents are also higher than the theoretical contents, but not as significantly as for the batch microgels. This is due to the fact that the comonomers were added afterward and consequently the VCL was able to polymerize beforehand.

In addition, the comonomer contents of BPMA‐L and BPMA‐LP microgels along with their copolymers could be determined via Raman spectroscopy due to their methacrylate groups. Therefore, Raman spectroscopy was performed using a calibration curve based on GMA.^[^ ^60^ ^]^ For the microgels and copolymers of BPMA‐L and BPMA‐LP, the carbonyl stretching vibrations of the methacrylate groups are visible at 1724 cm^−1^ and are adjacent to the VCL carbonyl stretching vibrations at 1636 cm^−1^ (Figures S4, S10, S7, and S11, Supporting Information). With increasing comonomer content, higher intensity is observed for the carbonyl stretching vibration band of the methacrylate group. The Raman spectra thus confirm identical contents for the BPMA‐L (Table S14, Supporting Information) and BPMA‐LP (Figure 4) in the microgels and copolymers.

### 2.4 Stimuli‐Responsive Studies of Microgels

**Figure 5:**
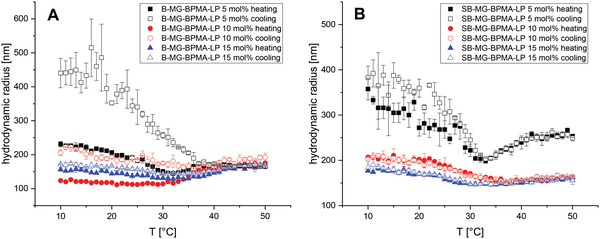
Hydrodynamic radii of A) B‐MG‐BPMA‐LP and B) SB‐MG‐BPMA‐LP microgels in water at temperatures between 10 and 50 °C measured via DLS.

The homopolymer of VCL has a lower critical solution temperature (LCST) at 32 °C,^[^ ^12^ ^]^ accordingly the microgels are expected to have a VPTT at this temperature. Hence, the hydrodynamic radii of the BPMA‐LP microgels were investigated via dynamic light scattering at temperatures between 10 and 50 °C in water (**Figure** **5**). Several microgels exhibit temperature‐responsive behavior and the hydrodynamic radii differ depending on the localization and content of the comonomer.

For the B‐MG‐BPMA‐LP microgels, the hydrodynamic radii are in the range of 120–210 nm at temperatures below the VPTT of PVCL with low polydispersity indices (PDIs) (Table S18, Supporting Information). Above this temperature, the hydrodynamic radii of the microgels appear to grow and persist during the cooling (Figure 5A). Yet also the PDIs increase with the higher temperatures, indicating the formation of agglomerate. The discrepancies of the microgel sizes between the heating and cooling cycles converge as the BPMA‐LP content increases. Similar observations can be made for the microgels in which the BPMA‐LP was incorporated in the corona. At 20 °C, the hydrodynamic radii are between 170 and 270 nm and larger compared to the batch microgels (Table S18, Supporting Information). The SB‐MGs with 10 mol% and 15 mol% BPMA‐LP collapse at higher temperatures, even though the PDIs (Table S18, Supporting Information) are increasing indicating the formation of agglomerates. Strong particle size increase was detected for the SB‐MG with the lowest BPMA‐LP content (Figure 5B). Still, the heating and cooling cycles show that the change in hydrodynamic radii of the microgels is reversible with temperature in contrast to microgels synthesized via batch polymerization.

The temperature‐responsive behavior of the microgels depends strongly on the distribution of the crosslink density within the microgels. Due to the different polymerization rates of cross‐linker and monomers, the microgel core exhibits a higher crosslinking density than the corona.^[^ ^64^, ^65^ ^]^ This results in the fact that the corona of the microgels has a greater influence on the temperature‐dependent swelling behavior of the microgels. The comonomer BPMA‐LP will also have a significant influence on the swelling behavior due to its hydrophobic properties. Since in the swollen state the Hamaker constant of the microgels matches that of the solvent (water), the interactions between microgels are governed by weak van der Waals attractive forces. Accordingly, the formation of aggregates in aqueous solution is suppressed. However, above the VPTT, the microgels collapse and the polymer segment density increases because water molecules are forced to leave the polymer network. This in turn leads to an increase in the Hamaker constant of the microgels, which is accompanied by an increase in van der Waals attractive forces.^[^ ^14^, ^66^ ^]^ Hydrophobic groups present in the polymer chains further enhance the attractive interactions and the probability of microgel agglomeration increases.^[^ ^14^, ^67^ ^]^ Cloud point studies and temperature‐dependent ^1^H‐NMR spectra were recorded to verify that the microgels are indeed in the collapsed state at higher temperatures.^[^ ^68^ ^]^ For this purpose, the microgels with the strongest increase in hydrodynamic radii above the VPTT (SB‐MG‐BPMA‐LP 5 mol%) were investigated. The cloud point analysis shows that below the VPTT the microgels are in a swollen state (transparent) and above the VPTT the solution starts to be turbid, indicating the collapsed state (Figure S9A, Supporting Information). As further confirmation of the collapsed state, temperature‐dependent ^1^H‐NMR studies were performed. Here, a change in the microgel microstructure due to the phase transition can be clearly identified (Figure S9B, Supporting Information). Between 20 and 30 °C, the proton signal intensities of PVCL polymer backbone remain unchanged. At 32 °C, the proton signal intensities of PVCL begin to faint and at 50 °C the PVCL signals are almost vanished. The remaining signals belong to the comonomer BPMA‐LP, which are also a little shifted due to the rising temperature. Thus, our experiments strongly indicate that the microgels are in a collapsed state above the VPTT. Consequently, an increase of the hydrodynamic radii above the VPTT measured by dynamic light scattering (DLS) advocates the formation of agglomerations.

Hence, we conclude that strong agglomeration of B‐MG‐BPMA‐LP microgels is probably the consequence of high crosslinking combined with hydrophobic interactions. In contrast, the SB‐MG‐BPMA‐LP microgels exhibit a lower crosslink density in the corona with the embedded BPMA‐LP. The hydrophobic domains appear to be weaker, leading to a reduced formation of agglomerates. In addition, the asymmetric shapes possibly influence the hydrodynamic radii leading to this deviation of the semibatch microgels.

The microgels synthesized from the comonomers PMA‐S, BPMA‐S, TPMA‐S, and BPMA‐L were also investigated for their temperature‐responsive behavior. Here, the hydrodynamic radii were measured at 20 and 50 °C to confirm an overall thermal responsiveness. This behavior was observed for all microgels that had VCL incorporated into them (Tables S3, S7, S11, and S15, Supporting Information). Only the microgels in which VCL was not incorporated showed no variation in hydrodynamic radii between 20 and 50 °C. However, the PDIs are low for all PMA‐S, BPMA‐S, TPMA‐S, and BPMA‐L microgels, indicating a narrow size distribution (Tables S3, S7, S11, and S15, Supporting Information).

In addition, we used ultraviolet–visible (UV–vis) spectroscopy to exclude potential overlaps between the used DLS laser wavelength (*λ* = 632.8 nm) and the absorption bands of the investigated microgels. Therefore, we selected monomer BPMA‐L and BPMA‐L based microgels with the highest BPMA‐L loading and recorded their UV–vis spectra (Figure S6, Supporting Information). These spectra indicate that absorption maximum for the BPMA‐L monomer and BPMA‐L based microgels located at 312 and 300 nm, respectively. This leads to the conclusion that the absorption bands of investigated compounds are out of range from the laser wavelength of the DLS instrument.

## 3 Conclusion

In conclusion, we developed computer simulation‐driven experimental synthesis approach to obtain microgels with various shapes based on the self‐assembly driven by supramolecular interactions followed by polymerization of tailored pyrazole‐modified monomers. Simulations predicted the formation of supramolecular aggregates from pyrazole‐modified monomers in dependence on their concentration, which can have a beneficial effect on the formation of anisotropic microgels and thus allowed a preselection of the monomers to be synthesized. Series of PVCL microgels with 5, 10, and 15 mol% of the respective pyrazole monomers were synthesized. By ^1^H‐NMR spectroscopy, *π*–*π* stacking of pyrazole monomers was confirmed, and the amounts of comonomer in the microgels were determined. The microscopy images revealed that for the microgels synthesized using semibatch addition mode, the morphology of the microgels varied from spherical to raspberry‐like or a dumbbell‐like with increasing BPMA‐LP content.

Our results demonstrate that the addition mode and concentration of tailored pyrazole‐modified comonomer allow to control the microgel morphology. In addition, the pyrazole monomers provide strong intermolecular *π*–*π* interactions, exhibit amphiphilic character, and long spacers between pyrazole group and polymerizable group to achieve the efficient morphology variation. This synthesis approach opens up new perspectives for the sustainable and upscalable synthesis of microgels with different shapes that can potentially find applications in interfacial catalysis, drug delivery and biosensing.

## 4 Experimental Section

### Chemicals

Acetonitrile (MeCN, Sigma Aldrich, Darmstadt, Germany, 99.8%), 2,2′‐azobis(2‐methylpropionamidine) dihydrochloride (AMPA, Sigma Aldrich, Darmstadt, Germany, 97%), benzyl benzoate (BB, Sigma Aldrich, Darmstadt, Germany, ≥99.0 %), *N,N*′‐methylenebis(acrylamide) (BIS, Sigma Aldrich, Darmstadt, Germany, 99%), *N‐*(bromomethyl)phthalimide (BrMPI, Sigma Aldrich, Darmstadt, Germany, 96%), 3‐bromo‐1‐propanol (Sigma Aldrich, Darmstadt, Germany, 97%), 2‐chloroethylamine hydrochloride (CEA, Sigma Aldrich, Darmstadt, Germany, 99%), chloroform (VWR Chemicals, Darmstadt, Germany), deuterated chloroform (CDCl_3_, Deutero GmbH, Kastellaun, Germany, 99.8%), dichloromethane (DCM, Sigma Aldrich, Darmstadt, Germany, >99.8%), 2,2‐dimethoxyethanamine (Sigma Aldrich, Darmstadt, Germany, 99%), ethanol (EtOH, VWR Chemicals, Darmstadt, Germany, 99.5%), hydrazine monohydrate (TCI Chemicals, Eschborn, Germany, >98%), MAC (Sigma Aldrich, Darmstadt, Germany, 97%), methanol (MeOH, Merck Millipore, Darmstadt, Germany, ≥99.8%), 1,8‐naphthalic anhydride (Sigma Aldrich, Darmstadt, Germany), potassium carbonate (Sigma Aldrich, Darmstadt, Germany, ≥99%), pyrazole (Sigma Aldrich, Darmstadt, Germany, 98%), sodium carbonate (VWR Chemicals, Darmstadt, Germany), sodium hydroxide (Merck Millipore, Darmstadt, Germany), sodium iodide (Sigma Aldrich, Darmstadt, Germany, ≥99 %), tetra‐*n*‐butylammonium bromide (TBAB, Sigma Aldrich, Darmstadt, Germany), toluene (Fisher Chemicals, Schwerte, Germany, ≥99.8%), *p*‐toluenesulfonic acid monohydrate (TCI, Eschborn, Germany, >98%), and triethylamine (TEA, Sigma Aldrich, Darmstadt, Germany, ≥99.5%) were used as received. VCL (Sigma Aldrich, Darmstadt, Germany, 98%) was distilled and recrystallized from hexane before use. GMA (Sigma Aldrich, Darmstadt, Germany, 97%) was purified via column chromatography on basic aluminum oxide. The concentration of the *n*‐butyllithium solution (*n*‐BuLi, Sigma Aldrich, 2.5 m in hexane) was titrimetically determined with 2‐hydroxybenzaldehyde phenylhydrazone (TCI Chemicals, Eschborn, Germany, >98.0%) before use. Tetrahydrofuran (THF) was dried through a solvent purification system SPS Compact (MBRAUN Inertgas‐Systeme GmbH, Garching, Germany).

### Synthesis of N‐(2‐(1H‐pyrazol‐1‐yl)ethyl)methacrylamide (PMA‐S)

PMA‐S was synthesized by functionalization of MAC with PEA. Therefore, PEA was synthesized according to literature (Supporting Information).^[^ ^54^ ^]^ The reaction was performed in DCM for 4 h at room temperature and the product was purified via column chromatography. Yield = 64%.

### Synthesis of N‐(2,2‐di(1H‐pyrazol‐1‐yl)ethyl)methacrylamide (BPMA‐S)

BPMA‐S was synthesized similarly to PMA‐S. The required BPEA was synthesized in a three‐step synthesis according to literature (Supporting Information).^[^ ^55^ ^]^ Yield = 66%.

### Synthesis of N‐(2,2,2‐tri(1H‐pyrazol‐1‐yl)ethyl)methacrylamide (TPMA‐S)

First, TPEA was prepared in a newly developed three‐step synthesis starting from the literature known synthesis of TPM (Supporting Information).^[^ ^56^ ^]^ Subsequently, the TPEA was functionalized to obtain TPMA‐S, which was prepared in the same manner as PMA‐S. Yield = 70%.

### Synthesis of 3‐((2,2‐Di(1H‐pyrazol‐1‐yl)ethyl)amino)propyl methacrylate (BPMA‐L)

To obtain BPMA‐L, the precursor 3‐bromopropyl methacrylate (BrPMA) was initially synthesized from 3‐bromo‐1‐propanol. Afterward, BrPMA was used to functionalize BPEA in the absence of oxygen to give BPMA‐L. Yield = 55%.

### Synthesis of 3‐((2,2‐Di(1H‐pyrazol‐1‐yl)ethyl)amino)‐2‐hydroxypropyl methacrylate (BPMA‐LP)

BPMA‐LP was synthesized by functionalization of glycidyl methacrylate with BPEA. The reaction was performed in methanol for 5 h at 60 °C and the product was purified via column chromatography. A mixture of *α*‐BPMA‐LP and *β*‐BPMA‐LP was obtained. Wherein, *α*‐BPMA‐LP was obtained as main product and *β*‐BPMA‐LP as by‐product. The product mixture was utilized for further microgel synthesis. Conversion = 54%.

### Synthesis of PVCL‐Based Microgels

All microgels based on poly(*N*‐vinylcaprolactam) were synthesized with 5, 10, and 15 mol% of the respective comonomer. *N,N′*‐methylenebis(acrylamide) was used as cross‐linker and 2,2′‐azobis(2‐methylpropionamidine) dihydrochloride was utilized as initiator. The comonomer‐rich core microgels were synthesized according to Häntzschel et al. using batch copolymerization procedure.^[^ ^59^ ^]^ For localization of the comonomer at the periphery of the microgels, the semibatch polymerization mode was used, where comonomer was added 3 min after the polymerization initiation according to Gau et al.^[^ ^60^ ^]^ The synthesized microgels were dialyzed against water for 5 d.

### Computer Simulations

For the all‐atom molecular dynamics simulation Gromacs 2019 package was used.^[^ ^69^ ^]^ The effect of pyrazole residues and their interaction with water in simulation are described by OPLS‐AA^[^ ^70^ ^]^ parameters with Lennard‐Jones and Coulomb interactions. Therefore, OPLS‐AA force field was applied for oligomer/monomer/methanol molecules and SPC/E^[^ ^71^ ^]^ for water. Periodic boundary conditions were used for a simulation box with the size of 10 nm × 10 nm × 10 nm for the monomers in the solvent and 13.5 nm × 12.1 nm × 14 nm for the surface adsorption simulations. The short‐range nonbonded interactions were calculated with cut‐off 1.2 nm and the long‐range electrostatic interactions by PME method.^[^ ^72^ ^]^ Bond vibrations, including the hydrogen atom, were constrained by the LINCS algorithm.^[^ ^73^ ^]^ To obtain an equilibrium state, two stages of simulations were implemented. The first short run was carried out in the *NPT* ensemble and a small time‐step of 0.01 fs with 10^6^ steps. Then all systems run with time‐step 2 fs up to 300 ns. The temperature was 300 K for all systems by the velocity‐rescale thermostat^[^ ^74^ ^]^ and a pressure of 1 atm for the *NPT* by the Berendsen barostat^[^ ^75^ ^]^ for the equilibration stage and the Parrinello–Rahman^[^ ^76^ ^]^ for the long run stage. The equilibrium state archives with the equilibration of the monomer adsorption on the PVCL surface. Thus, changes of the density profile for pyrazole residues were monitored. The Kullback–Leibler divergence^[^ ^77^ ^]^ was used as a method for a definition of the profile curve convergence to the equilibrium state (Figure S12, Supporting Information). The divergence between different stages of the equilibration was defined. Stages are 0–100, 100–150, 150–200, 200–250, 250–260, 260–270, 270–280, 280–290, and 290–300 ns. The divergence has a peak for some initial part of trajectories, which corresponds to a formation of a drop in water/methanol media before its adsorbing on the PVCL surface. The sequence of distributions approaches zero at last 50 ns for all trajectories which means a convergence to the equilibrium state. To evaluate the behavior of pyrazole‐rich residues in the solvent, all types of monomers in a water/methanol mixture were simulated with VCL monomers. Methanol concentration was taken to 2.2 vol% according to the experimental system. The resulting structures in both solvent and surface models are insoluble in mixture. This may be related to the non‐cosolvency effect of monomers in such mixture.^[^ ^78^ ^]^ The monomer number was *N* = 100 for all samples.

### NMR Spectroscopy

^1^H‐ and ^13^C‐NMR spectra were recorded on a Bruker AV400 Spectrometer (Bruker Corporation, Billerica, MA) at 400 and 100 MHz, respectively. These are indicated as follows: chemical shift *δ* (ppm) (multiplicity, number of protons, assignment, constituent). Chemical shifts were reported to the nearest 0.01 ppm for the ^1^H‐spectra and the nearest 0.1 ppm for the ^13^C‐spectra. Deuterated chloroform (CDCl_3_, *δ* H = 7.26 ppm) was used as solvent for all measurements. The comonomer content of the microgels and polymers was determined using benzyl benzoate as an internal standard. The temperature‐dependent studies were performed on a Bruker AV600 Spectrometer (Bruker Corporation, Billerica, MA) at 600 MHz. Deuterated water (D_2_O, *δ* H = 4.79 ppm) was used as a solvent and the sample concentration was 26.7 mg mL^−1^. The equilibration time was 15 min for each temperature step.

### Dynamic Light Scattering

DLS measurements were performed on an ALV/CGS‐3 Compact Goniometer System (ALV‐Laser Vertriebsgesellschaft mbH, Hessen, Germany) with an ALV/LSE 5004 Tau Digital Correlator and a JDS Uniphase laser for single temperature measurements. For the temperature trends, the measurements were performed on a Zetasizer Ultra XS (Malvern Panalytical Ltd., Malvern, UK). At both devices the laser operates at 632.8 nm and a scattering angle of 90° was used. The intensity time correlation functions were analyzed using CONTIN algorithm. All samples were filtered (1.2 µm PET filter, Chromafil) and diluted with fully demineralized water.

### Raman Spectroscopy

The Raman spectra of the microgels were measured on a Bruker FS 100/S Raman Spectrometer (Bruker Corporation, Billerica, MA) with a Nd:YAG laser (*λ* = 1064 nm). The measurements were performed with an energy of 200 mW, at a wavenumber between 400 and 3500 cm^−1^, resolution of 4 cm^−1^, and 1000 scans. For measuring, the microgels were lyophilized and pressed into an aluminum pan.

### Atomic Force Microscopy

The AFM images were recorded on a NanoScope V (Veeco Instruments, USA) with a NCH‐50 POINTPROBE‐Silicon SPM‐Sensor (Nanoworld, Neuchâtel, Switzerland) used as a cantilever. The measurements were performed with a resonance frequency of 320 kHz and a force constant of 42 N m^−1^. The diluted microgels were applied on a silicon wafer via dip‐coating and afterward measured in tapping mode. The recorded images were analyzed with the software Gwyddion 2.28.

### Electron Microscopy

TEM and cryo‐TEM images were recorded on a Libra 120 TEM (Zeiss, Oberkochen, Germany) with an acceleration voltage of 120 kV. SEM and STEM images were captured on an SU9000 ultrahigh resolution SEM (Hitachi High‐Technologies, Tokyo, Japan). The microgel samples were diluted to a concentration of 1 mg mL^−1^ and a single droplet was placed onto each carbon coated copper grid (CF400‐Cu) from Electron Microscopy Sciences. The microgel size of the recorded images was determined with the software ImageJ 1.52t.

### UV–Vis Spectroscopy

The UV–vis spectra were recorded on a JASCO V‐780 spectrophotometer (JASCO Deutschland GmbH, Pfungstadt, Germany) with a scan speed of 400 nm min^−1^ and a spectral resolution of 1 nm. The measurements of absorption were recorded in a wavelength range from 200 to 800 nm. Temperature‐dependent turbidity measurement was performed to determine the cloud point of the microgel in the range of 20–50 °C at a heating rate of 1 °C min^−1^ and at a wavelength of 500 nm. For all measurements, the samples were diluted 1:40 to ensure a maximum absorbance below 1.2.

## Conflict of Interest

The authors declare no conflict of interest.

## Author Contributions

F.G. planned and carried out the experiments. V.P. performed the computer simulations. F.G. wrote the manuscript with the support of V.P., A.P., I.P., and F.F. provided the substrate BPEA. D.E.D. helped in the detection of the *π*–*π* stacking via NMR. All authors reviewed the manuscript.

## Supporting information

Supporting Information

## Data Availability

The data that support the findings of this study are available in the supplementary material of this article.
