# Supplementary material for: Anisotropic Microgels by Supramolecular Assembly and Precipitation Polymerization of Pyrazole‐Modified Monomers
Source: Adv Sci (Weinh). 2022 Oct 30;9(36):2204853. doi: 10.1002/advs.202204853 (PMC9798967; doi:10.1002/advs.202204853)
Supplement: Supplementary file 1 — Supporting Information [file ADVS-9-2204853-s001.pdf]

ADVANCED SCIENCE
Open Access

Supporting Information

for *Adv. Sci.*, DOI 10.1002/advs.202204853

Anisotropic Microgels by Supramolecular Assembly and Precipitation Polymerization of Pyrazole-Modified Monomers

Frédéric Grabowski, Vladislav S. Petrovskii, Fabian Fink, Dan Eugen Demco, Sonja Herres-Pawlis, Igor I. Potemkin and Andrij Pich**

Supporting Information

Anisotropic Microgels by Supramolecular Assembly and Precipitation Polymerization of Pyrazole-Modified Monomers

Frédéric Grabowski, Vladislav S. Petrovskii, Fabian Fink, Dan Eugen Demco, Sonja Herres-Pawlis, Igor I. Potemkin, and Andrij Pich**

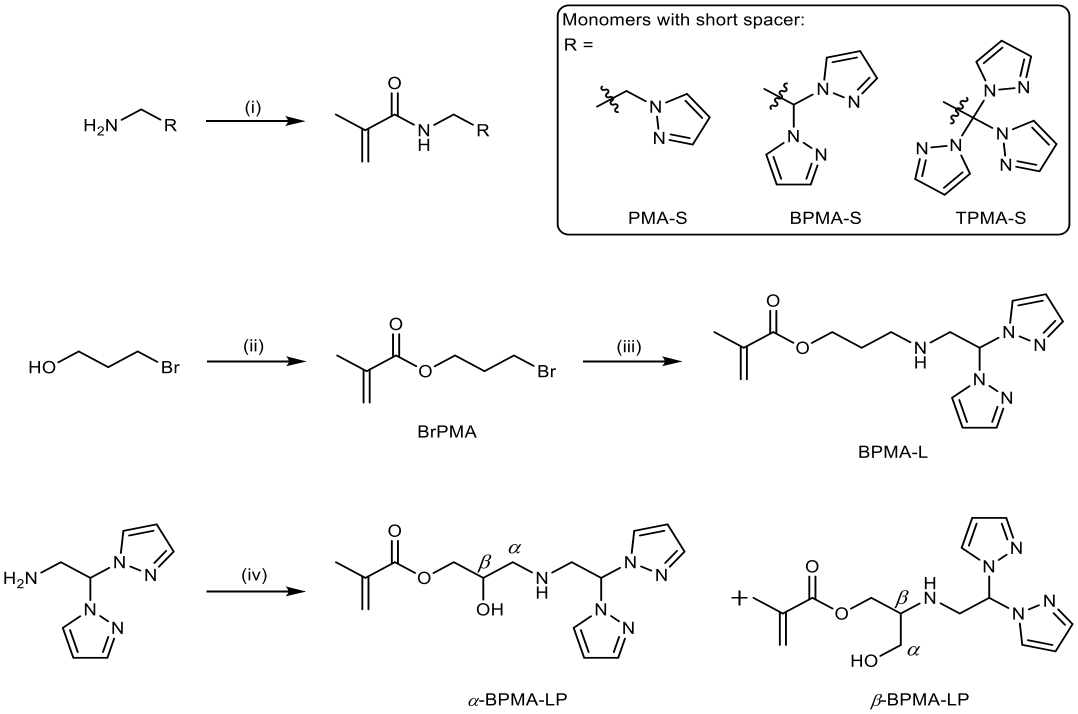

Scheme S1. Synthesis of monomers with different spacers and various numbers of pyrazole groups. Reagents and conditions: (i) MAC, TEA, DCM, 0 °C to rt, 5 h; (ii) MAC, TEA, DCM, 0 °C to rt, 24 h; (iii) BPEA, K_2CO_3, NaI, MeCN, 60 °C, 24 h; (iv) GMA, MeOH, 60 °C, 5 h. TEA = triethylamine, BrPMA = 3-bromopropyl methacrylate.

Synthesis of 2-(1*H*-pyrazol-1-yl)ethan-1-amine (pyrazolylethanamine, PEA)

The pyrazolylethanamine was synthesized according to literature with an additional purification step.[54] First, an acetonitrile fluid of 2-chloroethyl amine was prepared by

dissolving 20.0 g of 2-chloroethylamine (172.0 mmol) in MeCN (60 mL). The suspension was cooled to 0 °C and triethylamine (TEA, 23.8 mL, 172.0 mmol) was added dropwise over 30 min. The reaction stirred for 1 h at 0 °C. Afterwards, the white precipitate was removed by filtration and the desired fluid was obtained (yield = 100 %). In the next step, pyrazole (3.00 g, 44.1 mmol) was dissolved in MeCN (60 mL). Sodium hydroxide (5.29 g, 132.3 mmol) was added to this solution, which was then stirred for 30 min at room temperature. The reaction mixture was heated up to 75 °C and the acetonitrile fluid of 2-chloroethyl amine (88.2 mmol) was added dropwise over 30 min. Then, the solution was stirred at 75 °C overnight and after that, the solution was allowed to cool down to room temperature. The formed precipitate was removed by filtration and the solvent was removed *via* rotary evaporation. Finally, the product

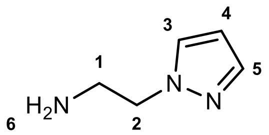

was condensed at 50 °C under high vacuum to remove residues resulting in a colorless oil (4.57 g, 92 %).

^{1}H-NMR (400 MHz, $CDCl_3$): δ [ppm] = 7.43 (d, *J* = 1.9 Hz, 1H, 5), 7.34 (d, *J* = 2.3 Hz, 1H, 3), 6.16 (t, *J* = 2.1 Hz, 1H, 4), 4.08 (t, *J* = 5.7 Hz, 2H, 2), 3.03 (t, *J* = 5.7 Hz, 2H, 1), 1.42 (br s, 2H, 6). ^{13}C-NMR (100 MHz, $CDCl_3$): δ [ppm] = 139.5 (1C, 5), 129.6 (1C, 3), 105.3 (1C, 4), 54.9 (1C, 2), 42.2 (1C, 1).

Synthesis of 2,2-di(1*H*-pyrazol-1-yl)ethan-1-amine (bis(pyrazolyl)ethanamine, BPEA)

The amine functionalized substrate was synthesized with minor modifications according to the procedure of Reger *et al.*[55] The product bis(pyrazolyl)ethanamine was synthesized starting from 1,8-naphthalic anhydride and 2,2-dimethoxyethanamine *via* a three-step synthesis. The crude product was obtained as a hygroscopic brown oil. After further purification *via* column chromatography on neutral silica gel (ethyl

acetate:methanol:triethylamine; 93:5:2, $R_f = 0.32$), the product was isolated as pale-orange crystals in 44% yield.

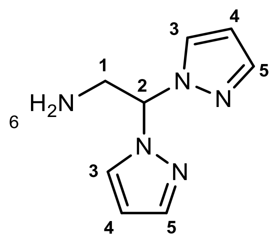

^{1}H-NMR (400 MHz, $CDCl_3$): δ [ppm] = 7.58 (d, J = 2.4 Hz, 2H, 3), 7.56 (d, J = 1.6 Hz, 2H, 5), 6.36 (t, J = 7.0 Hz, 1H, 2), 6.28 (t, J = 2.1 Hz, 2H, 4), 3.75 (d, J = 7.0 Hz, 2H, 1), 1.43 (br s, 2H, 6). ^{13}C-NMR (100 MHz, $CDCl_3$): δ [ppm] = 140.4 (2C, 5), 129.0 (2C, 3), 106.7 (2C, 4), 77.2 (1C, 2), 45.0 (1C, 1).

Synthesis of tri(1*H*-pyrazol-1-yl)methane (TPM)

The CH-acid substrate was synthesized according to the procedure of Reger *et al.*[56] The product tri(1*H*-pyrazol-1-yl)methane was prepared from pyrazole and chloroform. The

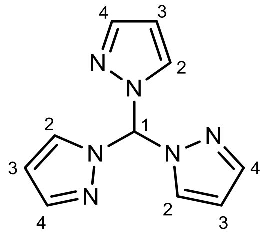

product was obtained as a pale-yellow solid in 62 % yield.

^{1}H-NMR (400 MHz, $CDCl_3$): δ [ppm] = 8.47 (s, 1H, 1), 7.67 (d, J = 1.8 Hz, 3H, 4), 7.58 (d, J = 2.6 Hz, 3H, 2), 6.36 (dd, J = 2.6, 1.8 Hz, 3H, 3). ^{13}C-NMR (100 MHz, $CDCl_3$): δ [ppm] = 141.9 (3C, 4), 129.7 (3C, 2), 107.4 (3C, 3), 83.3 (1C, 1).

Synthesis of 2-(2,2,2-tri(1*H*-pyrazol-1-yl)ethyl)isoindoline-1,3-dione (TPEID)

TPM (3.00 g, 14.0 mmol) was dissolved in THF (200 mL) and the solution was cooled to -78 °C. Under strong stirring, *n*-BuLi (6.7 mL, 16.8 mmol) was added dropwise. After 30 min, a solution consisting of *N*-(bromomethyl)phthalimide (4.9 g, 21.0 mmol) and THF (26 mL) was added dropwise and the mixture stirred for another hour at -78 °C. The reaction

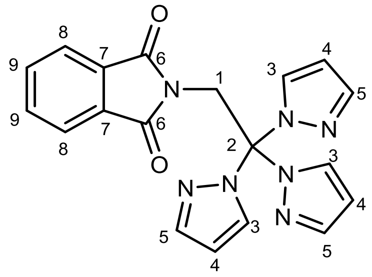

mixture was then gradually warmed to r.t. and stirred overnight. Next, H_2O (200 mL) was added and the mixture was extracted with Et_2O (3x 300 mL). The combined organic phases were dried over Na_2SO_4 and the solvent was removed *via* rotary evaporation. The crude product was recrystallized in EtOH and isolated product was maintained as white crystals (1.70 g, 33 %).

^{1}H-NMR (400 MHz, $CDCl_3$): δ [ppm] = 7.78 (dd, *J* = 5.5, 3.0 Hz, 2H, 8), 7.68 (dd, *J* = 5.5, 2.4 Hz, 2H, 9), 7.67 (d, *J* = 1.9 Hz, 3H, 5), 6.84 (d, *J* = 2.6 Hz, 3H, 3), 6.33 (dd, *J* = 2.7, 1.7 Hz, 3H, 4), 5.79 (s, 2H, 1). ^{13}C-NMR (100 MHz, $CDCl_3$): δ [ppm] = 167.5 (2C, 6), 141.7 (3C, 5), 134.0 (2C, 9), 132.2 (2C, 7), 130.6 (3C, 3), 123.6 (2C, 8), 107.4 (3C, 4), 89.6 (1C, 2), 43.6 (1C, 1).

Synthesis of 2,2,2-tri(1*H*-pyrazol-1-yl)ethan-1-amine (tris(pyrazolyl)ethanamine, TPEA)

TPEID (8.00 g, 21.4 mmol) and hydrazine monohydrate (8.30 mL, 171.2 mmol) were dissolved in toluene (300 mL). The reaction mixture was refluxed at 130 °C overnight, forming a yellow solid. After cooling to r.t., the toluene and excess hydrazine were removed *via* distillation. The solid was washed with hot acetone and filtered. The solvent from the

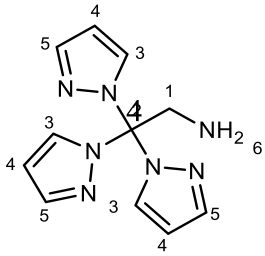

filtrate was removed *via* rotary evaporation. The crude product was purified *via* column chromatography on neutral silica gel (ethyl acetate:dichloromethane; 9:1, R_f = 0.32). The product was obtained as yellow oil (4.53 g, 87 %).

^{1}H-NMR (400 MHz, $CDCl_3$): δ [ppm] = 7.71 (d, *J* = 1.7 Hz, 3H, 5), 6.86 (d, *J* = 2.6 Hz, 3H, 3), 6.34 (dd, *J* = 2.7, 1.8 Hz, 3H, 4), 4.37 (s, 2H, 1), 2.70 (br s, 2H, 6). ^{13}C-NMR (100 MHz, $CDCl_3$): δ [ppm] = 142.0 (3C, 5), 130.0 (3C, 3), 106.9 (3C, 4), 91.0 (1C, 2), 52.0 (1C, 1).

Synthesis of *N*-(2-(1*H*-pyrazol-1-yl)ethyl)methacrylamide (pyrazolyl methacrylamide short, PMA-S)

PEA (2.50 g, 22.5 mmol) and TEA (6.23 mL, 45.0 mmol) were dissolved in DCM (90 mL) and cooled to 0 °C. Methacryloyl chloride (MAC, 4.70 g, 45.0 mmol) was dissolved in DCM (20 mL) and added dropwise to the reaction mixture. After 1 h, the mixture was warmed to r.t. and stirred for another 4 h. The solvent and TEA were removed *via* rotary evaporation. The crude product was purified *via* column chromatography on neutral silica gel (ethyl acetate:isopropanol; 9:1, R_f = 0.48). The product was obtained as a pale-yellow solid

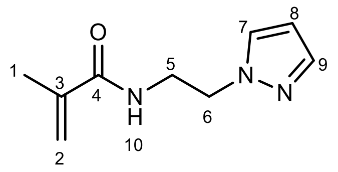

(2.60 g, 64 %).

^{1}H-NMR (400 MHz, $CDCl_3$): δ [ppm] = 7.54 (d, *J* = 1.9 Hz, 1H, 9), 7.38 (d, *J* = 2.3 Hz, 1H, 7), 6.56 (br s, 1H, 10), 6.26 (t, *J* = 2.1 Hz, 1H, 8), 5.66 (s, 1H, 2), 5.31 (s, 1H, 2), 4.30 (t, *J* = 5.4 Hz, 2H, 6), 3.74 (q, *J* = 5.6 Hz, 2H, 5), 1.92 (s, 3H, 1). ^{13}C-NMR (100 MHz, $CDCl_3$): δ [ppm] = 168.5 (1C, 4), 140.1 (1C, 9), 139.6 (1C, 3), 130.1 (1C, 7), 120.0 (1C, 2), 105.7 (1C, 8), 50.7 (1C, 6), 40.0 (1C, 5), 18.5 (1C, 1).

Synthesis of *N*-(2,2-di(1*H*-pyrazol-1-yl)ethyl)methacrylamide (bis(pyrazolyl) methacrylamide short, BPMA-S)

This comonomer was prepared in a similar manner as PMA-S, proceeding from BPEA

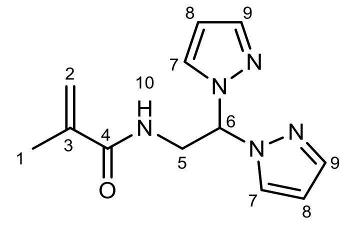

(2.50 g, 14.1 mmol), TEA (3.93 mL, 28.2 mmol) and MAC (2.86 g, 28.2 mmol). The crude product was purified *via* column chromatography on neutral silica gel (ethyl acetate:dichloromethane; 19:1, R_f = 0.55) resulting in a pale-yellow solid (2.30 g, 66 %).

^{1}H-NMR (400 MHz, $CDCl_3$): δ [ppm] = 7.59 (d, *J* = 1.8 Hz, 2H, 7), 7.57 (d, *J* = 2.4 Hz, 2H, 9), 6.69 (t, *J* = 6.9 Hz, 1H, 6), 6.40 (br s, 1H, 10), 6.29 (t, *J* = 2.2 Hz, 2H, 8), 5.66 (s, 1H, 2), 5.29 (s, 1H, 2), 4.40 (t, *J* = 6.6 Hz, 2H, 5), 1.87 (s, 3H, 1). ^{13}C-NMR (100 MHz, $CDCl_3$): δ [ppm] = 169.1 (1C, 4), 141.0 (2C, 9), 139.7 (1C, 3), 129.9 (2C, 7), 120.8 (1C, 2), 107.3 (2C, 8), 73.0 (1C, 6), 42.5 (1C, 5), 18.9 (1C, 1).

Synthesis of *N*-(2,2,2-tri(1*H*-pyrazol-1-yl)ethyl)methacrylamide (tris(pyrazolyl) methacrylamide short, TPMA-S)

This comonomer was prepared in a similar manner as PMA-S, proceeding from TPEA (4.52 g, 18.5 mmol), TEA (5.62 g, 55.5 mmol) and MAC (5.81 g, 55.5 mmol). After removing the solvent and TEA, the solid was washed with diethyl ether (4x 50 mL) followed by purification *via* column chromatography on neutral silica gel (hexane:ethyl acetate; 1:1,

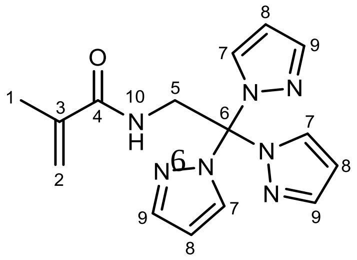

R_f = 0.39) resulting in a pale-yellow solid (4.01 g, 70 %).

^{1}H-NMR (400 MHz, $CDCl_3$): δ [ppm] = 8.08 (br s, 1H, 10), 7.68 (d, *J* = 1.8 Hz, 3H, 9), 7.06 (d, *J* = 2.6 Hz, 3H, 7), 6.35 (dd, *J* = 2.6, 1.8 Hz, 3H, 8), 5.61 (s, 1H, 2), 5.29 (s, 1H, 2), 5.09 (d, *J* = 6.1 Hz, 2H, 5), 1.89 (s, 3H, 1). ^{13}C-NMR (100 MHz, $CDCl_3$): δ [ppm] = 168.4 (1C, 4), 141.9 (3C, 9), 139.7 (1C, 3), 130.3 (3C, 7), 120.4 (1C, 2), 107.1 (3C, 8), 89.8 (1C, 6), 47.0 (1C, 5), 18.5 (1C, 1).

Synthesis of 3-bromopropyl methacrylate (BrPMA)

This compound was prepared in a similar manner as PMA-S, proceeding from 3-bromo-1-propanol (4.00 g, 28.8 mmol), TEA (12.0 mL, 86.4 mmol), MAC (9.03 g, 86.4 mmol) and reacted overnight. After removing the solvent and TEA, the solid was washed with diethyl ether (4x 25 mL) and later removed. The crude product was condensed at 50 °C under high vacuum and further purified *via* column chromatography on neutral silica gel

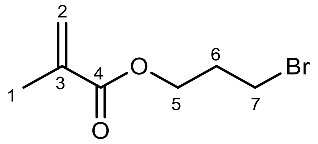

(hexane:ethyl acetate; 9:1, R_f = 0.40) resulting in a colorless liquid (4.53 g, 76 %).

^{1}H-NMR (400 MHz, $CDCl_3$): δ [ppm] = 6.09 (s, 1H, 2), 5.56 (s, 1H, 2), 4.28 (dt, *J* = 6.1, 4.9 Hz, 2H, 5), 3.62 (t, *J* = 6.4 Hz, 2H, 7, minor), 3.47 (t, *J* = 6.5 Hz, 2H, 7, major), 2.22 (quint, *J* = 6.3 Hz, 2H, 6, major), 2.13 (quint, *J* = 6.3 Hz, 2H, 6, minor), 1.93 (s, 3H, 1). ^{13}C-NMR (100 MHz, $CDCl_3$): δ [ppm] = 167.3 (1C, 4), 136.3 (1C, 3), 125.8 (1C, 2), 62.5 (1C, 5, major), 61.5 (1C, 5, minor), 41.4 (1C, 7, minor), 31.9 (1C, 6), 29.5 (1C, 7, major), 18.4 (1C, 1).

Synthesis of 3-((2,2-di(1*H*-pyrazol-1-yl)ethyl)amino)propyl methacrylate (bis(pyrazolyl) methacrylate long, BPMA-L)

A mixture of BrPMA (2.80 g, 13.5 mmol), BPEA (4.79 g, 27.0 mmol), K_2CO_3 (2.24 g, 16.2 mmol) and NaI (0.81 g, 5.4 mmol) in MeCN (150 mL) was placed in a 250 mL Schlenk flask. After the solution was degassed by five freeze-pump-thaw cycles, the reaction was performed at 60 °C for 24 h. The resulting mixture was filtered, and the precipitate was washed with MeCN (3x 20 mL). The solvent of the combined filtrate was removed *via* rotary evaporation and the crude product was purified *via* column chromatography on neutral silica gel (ethyl acetate:methanol; 19:1, R_f = 0.60) resulting in a viscous yellow oil (2.24 g, 55 %).

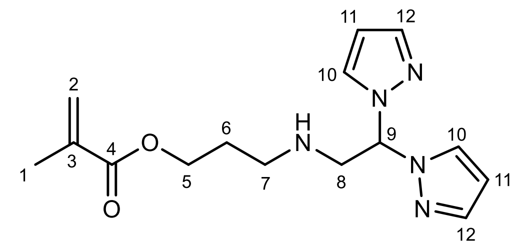

^{1}H-NMR (400 MHz, $CDCl_3$): δ [ppm] = 7.59 (d, *J* = 2.5 Hz, 2H, 10), 7.55 (d, *J* = 1.8 Hz, 2H, 12), 6.54 (t, *J* = 7.0 Hz, 1H, 9), 6.28 (t, *J* = 2.1 Hz, 2H, 11), 6.05 (s, 1H, 2), 5.54 (s, 1H, 2), 4.16 (t, *J* = 6.3 Hz, 2H, 5), 3.70 (d, *J* = 7.0 Hz, 2H, 8), 2.76 (t, *J* = 6.9 Hz, 2H, 7), 1.91 (s, 3H, 1), 1.81 (quint, *J* = 6.6 Hz, 2H, 6). ^{13}C-NMR (100 MHz, $CDCl_3$): δ [ppm] = 167.5 (1C, 4), 140.4 (2C, 12), 136.4 (1C, 3), 129.1 (2C, 10), 125.6 (1C, 2), 106.8 (2C, 11), 74.9 (1C, 9), 62.7 (1C, 5), 51.7 (1C, 8), 46.1 (1C, 7), 29.1 (1C, 6), 18.4 (1C, 1).

Synthesis of 3-((2,2-di(1*H*-pyrazol-1-yl)ethyl)amino)-2-hydroxypropyl methacrylate (bis(pyrazolyl) methacrylate long polar, BPMA-LP)

Glycidyl methacrylate (GMA, 0.300 g, 2.110 mmol, 1 eq.) and BPEA (0.449 g, 2.532 mmol, 1.2 eq.) were dissolved in MeOH (6 mL). The mixture reacted then for 5 h at 60 °C. After the reaction, the solvent was removed *via* rotary evaporation and the residue was

condensed under high vacuum to remove unreacted GMA. The product was further purified *via* column chromatography on neutral silica gel (ethyl acetate:methanol; 9:1, R_f = 0.70). A mixture of 3-((2,2-di(1*H*-pyrazol-1-yl)ethyl)amino)-2-hydroxypropyl methacrylate long polar (*α*-BPMA-LP) and 2-((2,2-di(1*H*-pyrazol-1-yl)ethyl)amino)-3-hydroxypropyl methacrylate long polar (*β*-BPMA-LP) with a ratio of 9:1 was obtained as a highly viscous orange oil (0.326 g, conversion = 54 %).

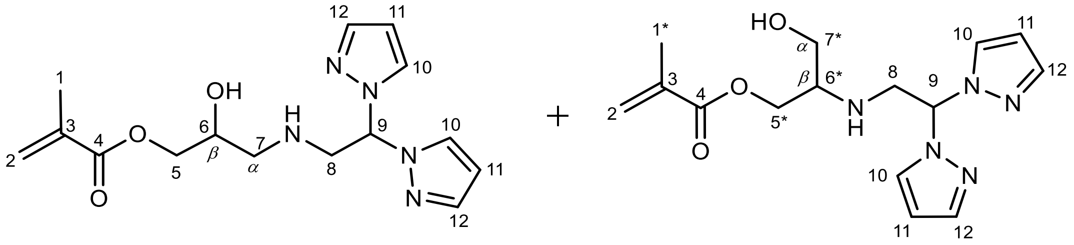

^{1}H-NMR (400 MHz, $CDCl_3$): δ [ppm] = 7.58 (d, *J* = 2.5 Hz, 2H, 10), 7.56 (d, *J* = 1.9 Hz, 2H, 12), 6.51 (t, *J* = 7.0 Hz, 1H, 9), 6.28 (t, *J* = 2.2 Hz, 2H, 11), 6.09 (s, 1H, 2), 5.57 (s, 1H, 2), 4.93 (quint, *J* = 4.6 Hz, 1H, 6*), 4.05-4.19 (m, 2H, 5), 3.87 (ddt, *J* = 8.1, 5.8, 4.1 Hz, 1H, 6), 3.64-3.80 (m, 4H, 5*+8), 2.93-3.06 (m, 2H, 7*), 2.62-2.88 (m, 2H, 7), 1.93 (s, 3H, 1), 1.84 (s, 3H, 1*). ^{13}C-NMR (100 MHz, $CDCl_3$): δ [ppm] = 167.5 (1C, 4), 140.5 (2C, 12), 136.0 (1C, 3), 129.2 (2C, 10), 126.2 (1C, 2), 107.0 (2C, 11), 74.7 (1C, 9), 73.2 (1C, 6*), 68.0 (1C, 6), 66.6 (1C, 5), 63.6 (1C, 5*), 51.7 (1C, 8), 51,4 (1C, 7), 49.9 (1C, 7*), 20.4 (1C, 1*), 18.4 (1C, 1).

Synthesis of PMA-S Microgels

The microgels with various PMA-S contents in the core were synthesized using batch free radical precipitation polymerization according to Häntzschel *et al.*[59] VCL (amounts see **Table S1**) and BIS (0.285 mmol, 3 mol%) were dissolved in water (131 mL). PMA-S (amounts see **Table S1**) was first dissolved in methanol (2.2 vol%) and then added to the solution. The solution was purged with nitrogen for 1 h at 70 °C. Following, a solution

consisting of AMPA (0.076 mmol, 0.8 mol%) and water (4 mL) was added to initiate the polymerization. Then, the mixture was stirred for 2 h at 70 °C. The obtained microgels were dialyzed against deionized water (MWCO: 12.000-14.000 Da) for 5 days.

For the microgels containing comonomer at the periphery, the synthesis was carried out according to Gau *et al.* using semi-batch free radical precipitation polymerization.[60] Similarly to the batch synthesis, VCL (amounts see **Table S1**) and BIS (0.285 mmol, 3 mol%) were dissolved in water (131 mL). Afterwards, the solution was purged with nitrogen for 1 h at 70 °C under strong stirring. For initiation, AMPA (20.6 mg, 0.076 mmol, 0.8 mol%) was dissolved in water (4 mL) and added to the reaction mixture. Subsequently, a solution of PMA-S (amounts see **Table S1**) and methanol (2.2 vol%) was added 3 min after the initiation.

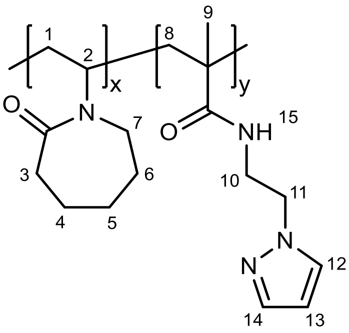

The mixture was then stirred for 2 h at 70 °C. The same purification was performed as for the batch microgels.

^{1}H-NMR (400 MHz, $CDCl_3$) of B-MG-PMA-S 5 mol%: δ [ppm] = 7.62 (1H, 14), 7.49 (1H, 12), 6.22 (1H, 13), 4.06-4.81 (3H, 2+11), 3.52-3.89 (2H, 10), 2.83-3.47 (2H, 7), 2.12-2.78 (2H, 3), 0.68-2.06 (13H, 1+4+5+6+8+9).

^{1}H-NMR (400 MHz, $CDCl_3$) of B-MG-PMA-S MG 10 mol%: δ [ppm] = 7.61 (1H, 14), 7.49 (1H, 12), 6.22 (1H, 13), 4.01-4.84 (3H, 2+11), 3.49-3.89 (2H, 10), 2.83-3.44 (2H, 7), 2.39 (2H, 3), 0.63-2.06 (13H, 1+4+5+6+8+9).

^{1}H-NMR (400 MHz, $CDCl_3$) of B-MG-PMA-S 15 mol%: δ [ppm] = 7.46 (1H, 12), 6.21 (1H, 13), 4.21 (2H, 11), 3.55 (2H, 10), 2.00 (2H, 8), 1.75 (3H, 9).

^{1}H-NMR (400 MHz, $CDCl_3$) of SB-MG-PMA-S 5 mol%: δ [ppm] = 7.63 (1H, 14), 7.49 (1H, 12), 6.22 (1H, 13), 4.05-4.79 (3H, 2+11), 2.85-3.51 (2H, 7), 2.01-2.75 (2H, 3), 0.70-1.98 (13H, 1+4+5+6+8+9).

^{1}H-NMR (400 MHz, $CDCl_3$) of SB-MG-PMA-S 10 mol%: δ [ppm] = 7.61 (1H, 14), 7.49 (1H, 12), 6.22 (1H, 13), 4.06-4.77 (3H, 2+11), 3.51-3.91 (2H, 10), 2.83-3.47 (2H, 7), 1.94-2.71 (2H, 3), 0.74-1.93 (13H, 1+4+5+6+8+9).

^{1}H-NMR (400 MHz, $CDCl_3$) of SB-MG-PMA-S 15 mol%: δ [ppm] = 7.60 (1H, 14), 7.49 (1H, 12), 6.21 (1H, 13), 4.06-4.79 (3H, 2+11), 3.51-3.87 (2H, 10), 2.81-3.44 (2H, 7), 2.04-2.73 (2H, 3), 0.69-2.03 (13H, 1+4+5+6+8+9).

Table S1. Amounts of used VCL and PMA-S for microgel synthesis and the corresponding gravimetrically determined yields.

sample	m(VCL) [g]	m(PMA-S) [g]	yield [%]
B-MG-PMA-S 5 mol%	1.256	0.085	37
B-MG-PMA-S 10 mol%	1.192	0.171	25
B-MG-PMA-S 15 mol%	1.125	0.255	0.4
SB-MG-PMA-S 5 mol%	1.257	0.085	77
SB-MG-PMA-S 10 mol%	1.190	0.171	57
SB-MG-PMA-S 15 mol%	1.125	0.256	76

Table S2. Theoretical PMA-S content and determined PMA-S content of microgels and copolymer *via* ^{1}H-NMR.

sample	$PMA\text{-}S_{theor}$ [mol%]	$PMA\text{-}S_{NMR}$ [mol%]
B-MG-PMA-S 5 mol%	5.0	12.3
B-MG-PMA-S 10 mol%	10.0	46.6
B-MG-PMA-S 15 mol%	15.0	100.0
SB-MG-PMA-S 5 mol%	5.0	9.2
SB-MG-PMA-S 10 mol%	10.0	22.6
SB-MG-PMA-S 15 mol%	15.0	35.1
$P(VCL)_{0.85}\text{-}(PMA\text{-}S)_{0.15}$	15.0	50.0

Table S3. Hydrodynamic radii and PDI values of PMA-S microgels at 20 °C and 50 °C determined *via* DLS.

sample	R_h 20 °C [nm]	PDI 20 °C	R_h 50 °C [nm]	PDI 50 °C
B-MG-PMA-S 5 mol%	175.8 ± 6.7	0.033	131.0 ± 2.1	0.076
B-MG-PMA-S 10 mol%	143.8 ± 5.1	0.041	138.4 ± 0.5	0.018
B-MG-PMA-S 15 mol%	138.1 ± 1.9	0.006	137.4 ± 1.5	0.025
SB-MG-PMA-S 5 mol%	275.6 ± 12.2	0.044	137.9 ± 0.7	0.095
SB-MG-PMA-S 10 mol%	225.1 ± 8.3	0.091	141.7 ± 1.9	0.001
SB-MG-PMA-S 15 mol%	275.3 ± 14.1	0.089	153.1 ± 2.3	0.013

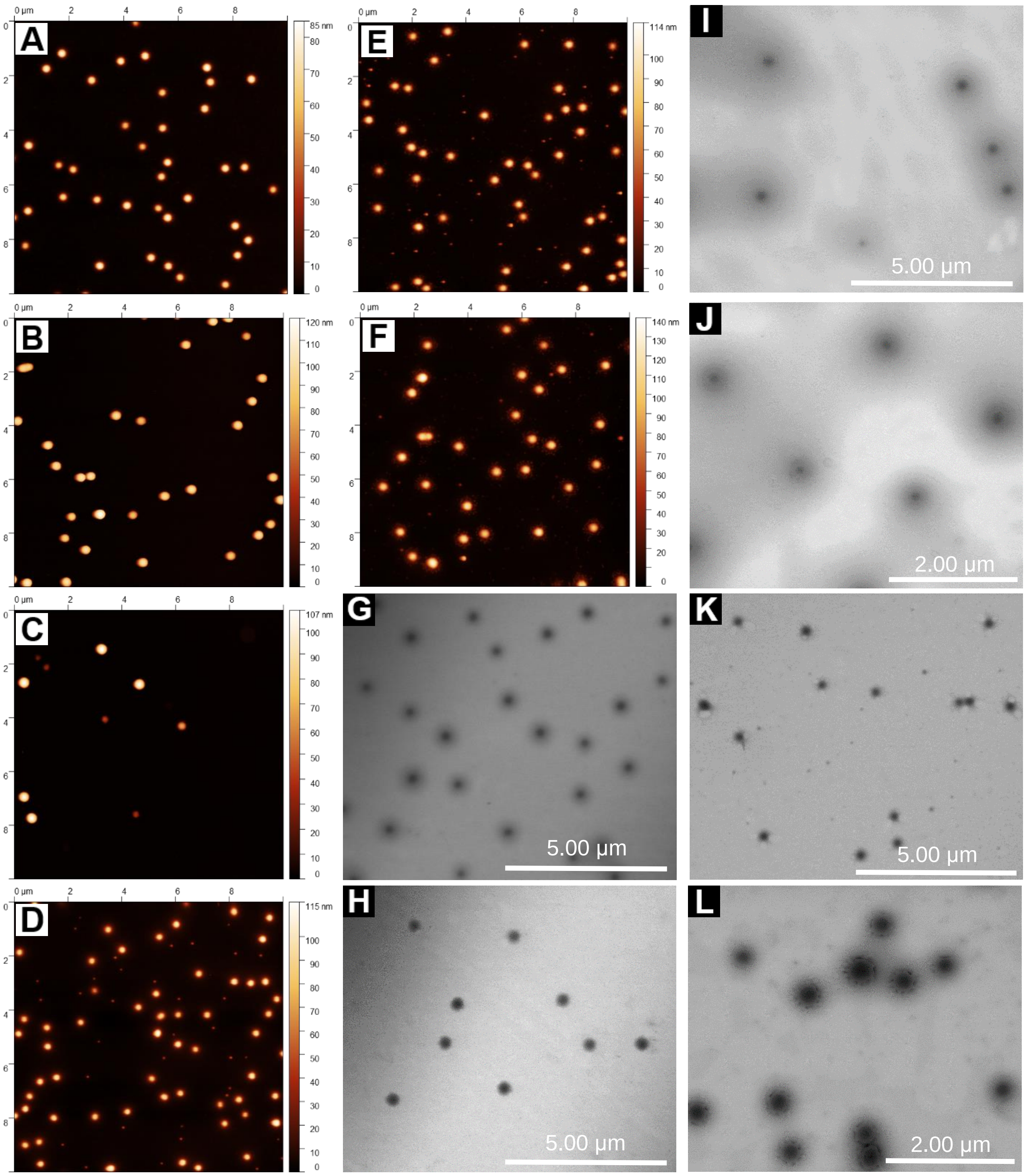

Figure S1. AFM (A-F) and STEM (G-L) images of the PMA-S microgels. The batch microgels with 5 mol% (A & G), 10 mol% (B & H) and 15 mol% (C & I) are ordered by increasing content of PMA-S for the respective microscopy method. The same applies for the semi-batch microgels with 5 mol% (D & J), 10 mol% (E & K) and 15 mol% (F & L).

Table S4. Diameter of the B-MG- and SB-MG-PMA-S microgels determined *via* STEM images.

sample	diameter [nm]
B-MG-PMA-S 5 mol%	610.4 ± 34.2
B-MG-PMA-S 10 mol%	424.7 ± 31.4
B-MG-PMA-S 15 mol%	350.0 ± 22.4
SB-MG-PMA-S 5 mol%	366.1 ± 50.6
SB-MG-PMA-S 10 mol%	288.0 ± 28.5
SB-MG-PMA-S 15 mol%	317.7 ± 26.9

Synthesis of BPMA-S Microgels

The core-shell microgels were prepared in a similar manner to the PMA-S microgels, proceeding from VCL (amounts see **Table S5**), BIS (0.285 mmol, 3 mol%), BPMA-S

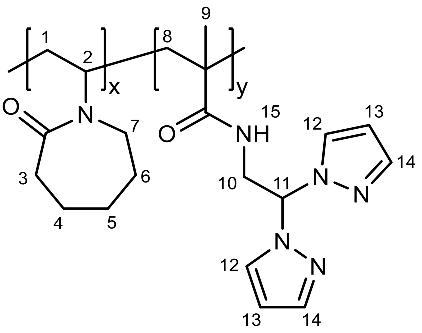

(amounts see **Table S5**) and AMPA (0.076 mmol, 0.8 mol%).

^{1}H-NMR (400 MHz, $CDCl_3$) of B-MG-BPMA-S 5 mol%: δ [ppm] = 7.56 (2H, 14), 6.26 (2H, 13), 4.13-4.78 (3H, 2+10), 2.89-3.48 (2H, 7), 2.18-2.77 (2H, 3), 0.68-2.12 (13H, 1+4+5+6+8+9).

^{1}H-NMR (400 MHz, $CDCl_3$) of B-MG-BPMA-S 10 mol%: δ [ppm] = 7.76 (2H, 12), 7.55 (2H, 14), 6.83 (1H, 11), 6.25 (2H, 13), 4.04-4.93 (3H, 2+10), 2.86-3.47 (2H, 7), 2.24-2.73 (2H, 3), 0.65-2.14 (13H, 1+4+5+6+8+9).

^{1}H-NMR (400 MHz, $CDCl_3$) of B-MG-BPMA-S 15 mol%: δ [ppm] = 7.34-8.04 (4H, 12+14), 6.25 (2H, 13), 4.26 (2H, 10), 2.30 (2H, 8), 2.00 (3H, 9).

^{1}H-NMR (400 MHz, $CDCl_3$) of SB-MG-BPMA-S 5 mol%: δ [ppm] = 7.81 (2H, 12), 7.55 (2H, 14), 6.26 (2H, 13), 4.07-4.84 (3H, 2+10), 2.91-3.47 (2H, 7), 2.19-2.74 (2H, 3), 0.74-2.12 (13H, 1+4+5+6+8+9).

^{1}H-NMR (400 MHz, $CDCl_3$) of SB-MG-BPMA-S 10 mol%: δ [ppm] = 7.77 (2H, 12), 7.55 (2H, 14), 6.83 (1H, 11), 6.25 (2H, 13), 4.05-4.82 (3H, 2+10), 2.88-3.47 (2H, 7), 2.22-2.73 (2H, 3), 0.62-2.13 (13H, 1+4+5+6+8+9).

^{1}H-NMR (400 MHz, $CDCl_3$) of SB-MG-BPMA-S 15 mol%: δ [ppm] = 7.77 (2H, 12), 7.55 (2H, 14), 6.83 (1H, 11), 6.25 (2H, 13), 3.98-4.76 (3H, 2+10), 2.87-3.43 (2H, 7), 2.20-2.71 (2H, 3), 0.54-2.09 (13H, 1+4+5+6+8+9).

Table S5. Amounts of used VCL and BPMA-S for microgel synthesis and the corresponding gravimetrically determined yields.

sample	m(VCL) [g]	m(BPMA-S) [g]	yield [%]
B-MG-BPMA-S 5 mol%	1.256	0.117	54
B-MG-BPMA-S 10 mol%	1.190	0.233	70
B-MG-BPMA-S 15 mol%	1.124	0.350	23
SB-MG-BPMA-S 5 mol%	1.256	0.117	74
SB-MG-BPMA-S 10 mol%	1.190	0.233	70
SB-MG-BPMA-S 15 mol%	1.124	0.350	56

Table S6. Theoretical BPMA-S content and determined BPMA-S content of microgels and copolymer *via* ^{1}H-NMR.

sample	$\text{BPMA-S}_{\text{theor}}$ [mol%]	$\text{BPMA-S}_{\text{NMR}}$ [mol%]
B-MG-BPMA-S 5 mol%	5.0	0.5
B-MG-BPMA-S 10 mol%	10.0	5.2
B-MG-BPMA-S 15 mol%	15.0	100.0
SB-MG-BPMA-S 5 mol%	5.0	1.9
SB-MG-BPMA-S 10 mol%	10.0	12.8
SB-MG-BPMA-S 15 mol%	15.0	29.1
$P(VCL)_{0.85}$-$(BPMA\text{-}S)_{0.15}$	15.0	41.0

Table S7. Hydrodynamic radii and PDI values of BPMA-S microgels at 20 °C and 50 °C determined *via* DLS.

sample	R_h 20 °C [nm]	PDI 20 °C	R_h 50 °C [nm]	PDI 50 °C
B-MG-BPMA-S 5 mol%	204.4 ± 4.4	0.022	128.8 ± 1.1	0.004
B-MG-BPMA-S 10 mol%	140.2 ± 5.5	0.029	115.7 ± 1.5	0.035
B-MG-BPMA-S 15 mol%	142.7 ± 1.9	0.275	150.0 ± 0.9	0.177
SB-MG-BPMA-S 5 mol%	258.7 ± 7.6	0.062	146.5 ± 0.1	0.044
SB-MG-BPMA-S 10 mol%	161.3 ± 5.6	0.016	132.6 ± 3.3	0.088
SB-MG-BPMA-S 15 mol%	147.4 ± 6.0	0.032	134.1 ± 0.3	0.006

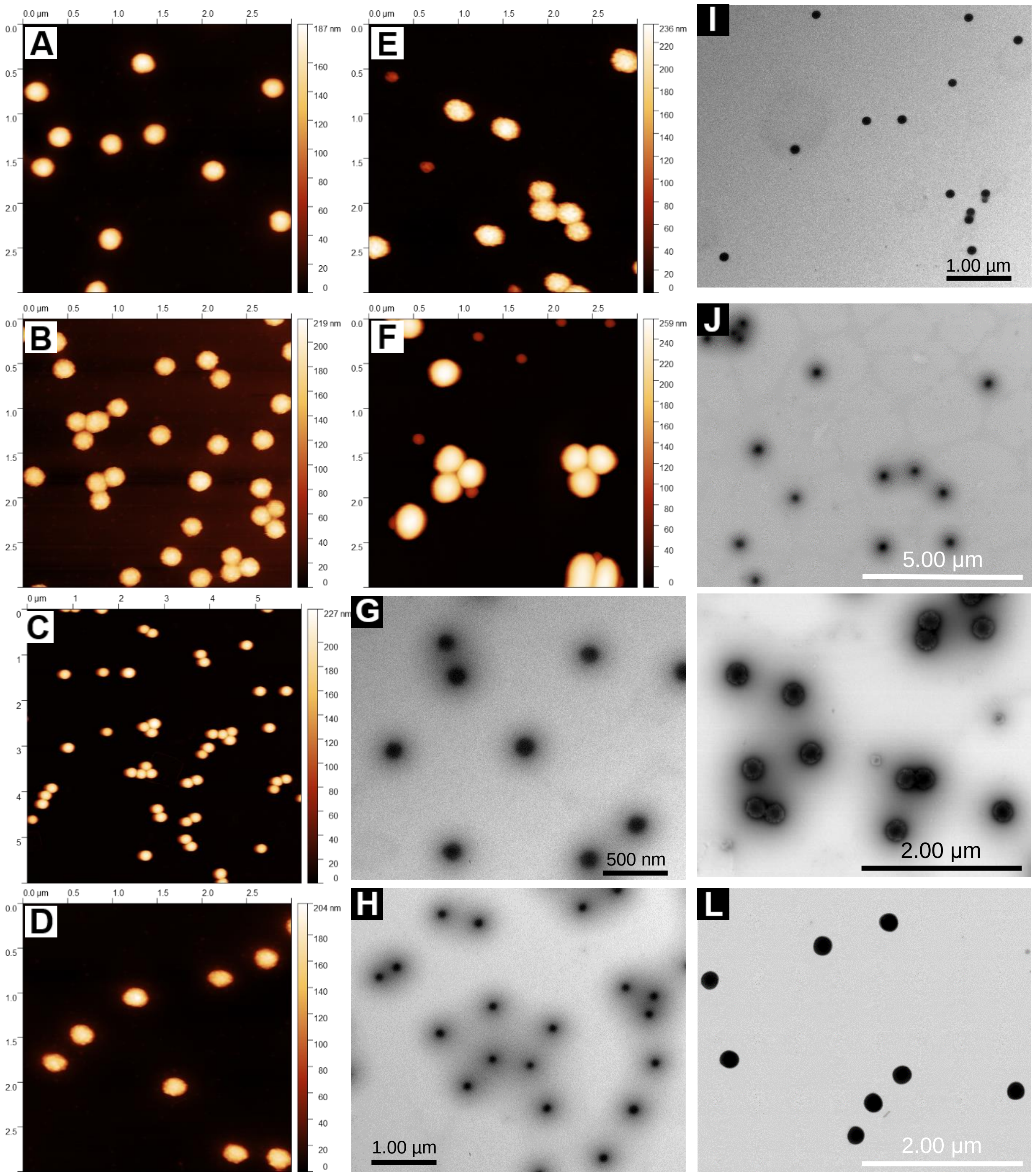

Figure S2. AFM (A-F) and STEM/TEM (G-L) images of the BPMA-S microgels. The batch microgels with 5 mol% (A & G), 10 mol% (B & H) and 15 mol% (C & I) are ordered by increasing content of BPMA-S for the respective microscopy method. The same applies for the semi-batch microgels with 5 mol% (D & J), 10 mol% (E & K) and 15 mol% (F & L).

Table S8. Diameter of the B-MG- and SB-MG-BPMA-S microgels determined *via* STEM/TEM images.

sample	diameter [nm]
B-MG-BPMA-S 5 mol%	246.2 ± 20.5
B-MG-BPMA-S 10 mol%	265.2 ± 21.4
B-MG-BPMA-S 15 mol%	132.0 ± 10.9
SB-MG-BPMA-S 5 mol%	567.2 ± 44.9
SB-MG-BPMA-S 10 mol%	249.3 ± 23.0
SB-MG-BPMA-S 15 mol%	218.8 ± 8.4

Synthesis of TPMA-S Microgels

The core-shell microgels were prepared in a similar manner to the PMA-S microgels, proceeding from VCL (amounts see **Table S9**), BIS (0.285 mmol, 3 mol%), TPMA-S

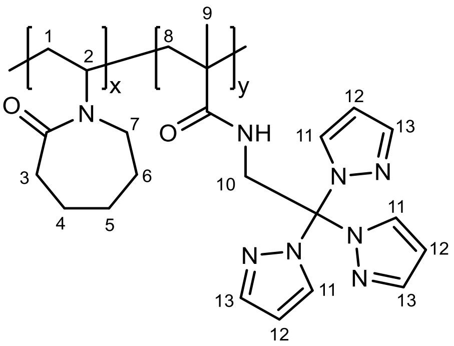

(amounts see **Table S9**) and AMPA (0.076 mmol, 0.8 mol%).

^{1}H-NMR (400 MHz, $CDCl_3$) of B-MG-TPMA-S 5 mol%: δ [ppm] = 7.67 (3H, 13), 6.95 (3H, 11), 6.33 (3H, 12), 4.12-4.85 (1H, 2), 2.93-3.47 (2H, 7), 2.16-2.90 (2H, 3), 0.60-2.08 (13H, 1+4+5+6+8+9).

^{1}H-NMR (400 MHz, $CDCl_3$) of B-MG-TPMA-S 10 mol%: δ [ppm] = 7.64 (3H, 13), 6.93 (3H, 11), 6.30 (3H, 12), 4.20-5.49 (2H, 10), 0.28-3.28 (5H, 8+9).

^{1}H-NMR (400 MHz, $CDCl_3$) of B-MG-TPMA-S 15 mol%: δ [ppm] = 7.64 (3H, 13), 6.93 (3H, 11), 6.27 (3H, 12), 4.23-5.52 (2H, 10), 1.14-3.14 (2H, 8), 0.21-1.02 (3H, 9).

^{1}H-NMR (400 MHz, $CDCl_3$) of SB-MG-TPMA-S 5 mol%: δ [ppm] = 7.66 (3H, 13), 6.98 (3H, 11), 6.33 (3H, 12), 4.09-4.81 (1H, 2), 2.89-3.54 (2H, 7), 2.15-2.78 (2H, 3), 0.70-2.10 (13H, 1+4+5+6+8+9).

^{1}H-NMR (400 MHz, $CDCl_3$) of SB-MG-TPMA-S 10 mol%: δ [ppm] = 7.66 (3H, 13), 6.99 (3H, 11), 6.32 (3H, 12), 4.80-5.31 (2H, 10), 4.13-4.72 (1H, 2), 2.90-3.44 (2H, 7), 2.14-2.71 (2H, 3), 0.64-2.06 (13H, 1+4+5+6+8+9).

^{1}H-NMR (400 MHz, $CDCl_3$) of SB-MG-TPMA-S 15 mol%: δ [ppm] = 7.65 (3H, 13), 7.00 (3H, 11), 6.32 (3H, 12), 4.80-5.37 (2H, 10), 4.13-4.70 (1H, 2), 2.84-3.46 (2H, 7), 2.11-2.68 (2H, 3), 0.47-2.07 (13H, 1+4+5+6+8+9).

Table S9. Amounts of used VCL and TPMA-S for microgel synthesis and the corresponding gravimetrically determined yields.

**sample**	**m(VCL) [g]**	**m(TPMA-S) [g]**	**yield [%]**
B-MG-TPMA-S 5 mol%	1.257	0.148	23
B-MG-TPMA-S 10 mol%	1.191	0.296	20
B-MG-TPMA-S 15 mol%	1.125	0.444	25
SB-MG-TPMA-S 5 mol%	1.257	0.148	61
SB-MG-TPMA-S 10 mol%	1.191	0.296	53
SB-MG-TPMA-S 15 mol%	1.125	0.444	48

Table S10. Theoretical TPMA-S content and determined TPMA-S content of microgels and copolymer *via* ^{1}H-NMR.

**sample**	**TPMA-S$_{theor}$ [mol%]**	**TPMA-S$_{NMR}$ [mol%]**
B-MG-TPMA-S 5 mol%	5.0	11.9
B-MG-TPMA-S 10 mol%	10.0	100
B-MG-TPMA-S 15 mol%	15.0	100
SB-MG-TPMA-S 5 mol%	5.0	4.9
SB-MG-TPMA-S 10 mol%	10.0	15.6
SB-MG-TPMA-S 15 mol%	15.0	29.3
$P(VCL)_{0.85}$-$(TPMA\text{-}S)_{0.15}$	15.0	36.0

Table S11. Hydrodynamic radii and PDI values of TPMA-S microgels at 20 °C and 50 °C determined *via* DLS.

sample	R_h 20 °C [nm]	PDI 20 °C	R_h 50 °C [nm]	PDI 50 °C
B-MG-TPMA-S 5 mol%	263.8 ± 0.9	0.306	733.7 ± 17.6	0.161
B-MG-TPMA-S 10 mol%	103.4 ± 3.3	0.352	116.0 ± 1.6	0.370
B-MG-TPMA-S 15 mol%	115.6 ± 5.3	0.414	124.4 ± 0.8	0.310
SB-MG-TPMA-S 5 mol%	180.7 ± 6.3	0.086	131.4 ± 2.3	0.076
SB-MG-TPMA-S 10 mol%	133.8 ± 4.3	0.037	128.9 ± 1.3	0.010
SB-MG-TPMA-S 15 mol%	124.7 ± 4.2	0.040	326.7 ± 43.4	0.210

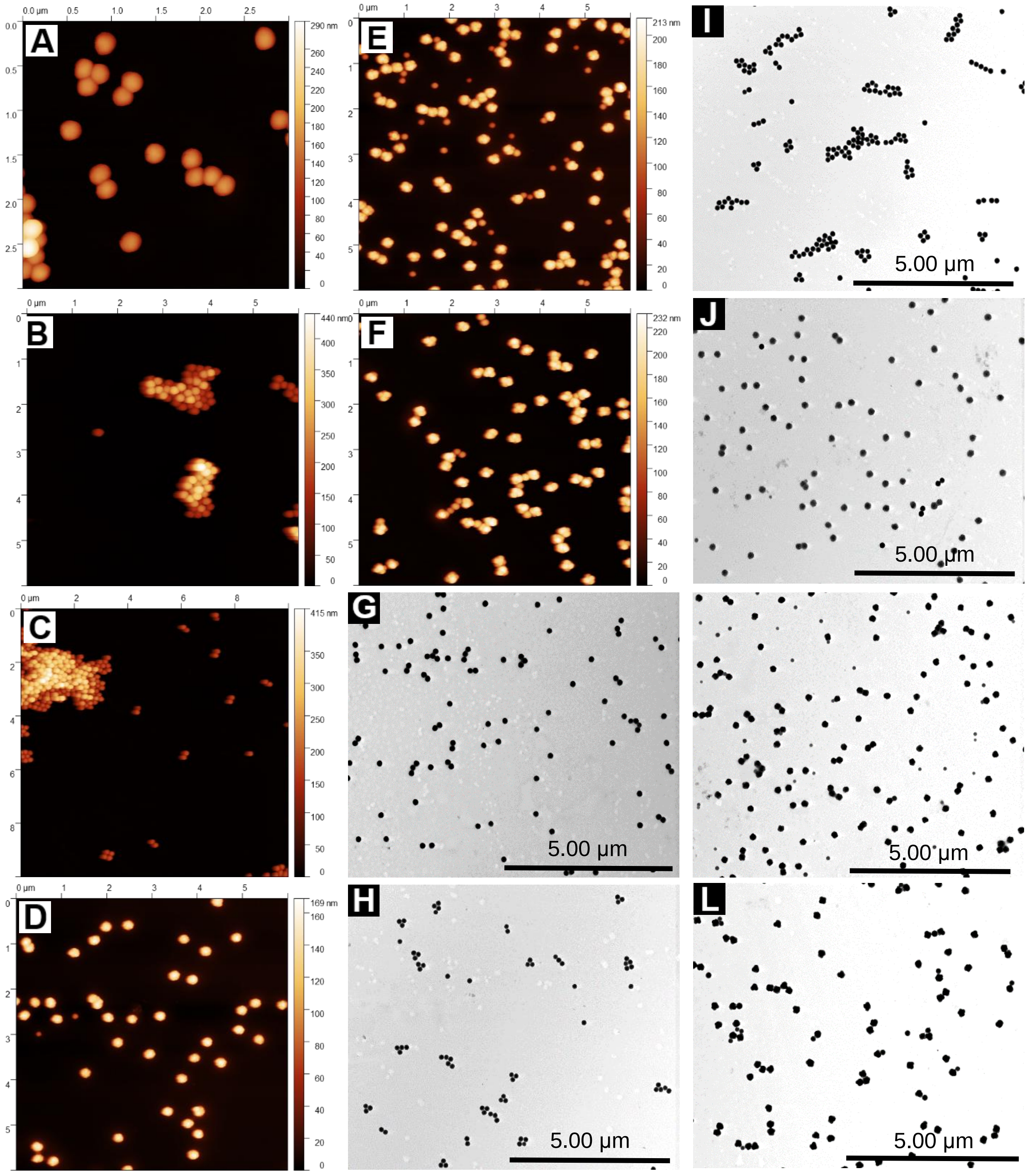

Figure S3. AFM (A-F) and STEM (G-L) images of the TPMA-S microgels. The batch microgels with 5 mol% (A & G), 10 mol% (B & H) and 15 mol% (C & I) are ordered by increasing content of TPMA-S for the respective microscopy method. The same applies for the semi-batch microgels with 5 mol% (D & J), 10 mol% (E & K) and 15 mol% (F & L).

Table S12. Diameter of the B-MG- and SB-MG-TPMA-S microgels determined *via* STEM images.

sample	diameter [nm]
B-MG-TPMA-S 5 mol%	137.8 ± 14.3
B-MG-TPMA-S 10 mol%	119.8 ± 7.0
B-MG-TPMA-S 15 mol%	133.6 ± 8.9
SB-MG-TPMA-S 5 mol%	175.5 ± 9.9
SB-MG-TPMA-S 10 mol%	167.5 ± 16.3
SB-MG-TPMA-S 15 mol%	186.7 ± 24.0

Synthesis of BPMA-L Microgels

The core-shell microgels were prepared in a similar manner to the PMA-S microgels, proceeding from VCL (amounts see **Table S13**), BIS (0.285 mmol, 3 mol%), BPMA-L

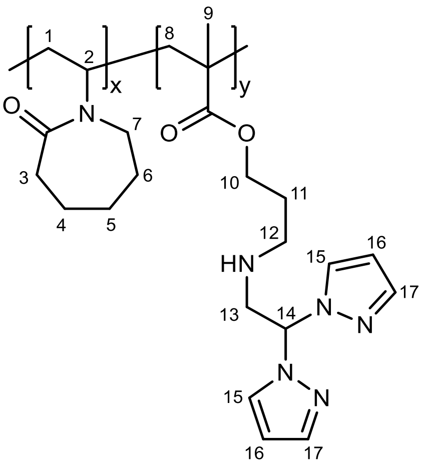

(amounts see **Table S13**) and AMPA (0.076 mmol, 0.8 mol%).

^{1}H-NMR (400 MHz, $CDCl_3$) of B-MG-BPMA-L 5 mol%: δ [ppm] = 7.66 (2H, 15), 7.52 (2H, 17), 6.24 (2H, 16), 4.16-4.78 (3H, 2+10), 3.69 (2H, 13), 2.86-3.48 (2H, 7), 2.13-2.63 (2H, 3), 0.67-2.07 (15H, 1+4+5+6+8+9+11).

^{1}H-NMR (400 MHz, $CDCl_3$) of B-MG-BPMA-L 10 mol%: δ [ppm] = 7.63 (2H, 15), 7.52 (2H, 17), 6.59 (1H, 14), 6.24 (2H, 16), 4.14-4.83 (3H, 2+10), 3.67 (2H, 13), 2.87-3.48 (2H, 7), 2.66 (2H, 12), 2.11-2.62 (2H, 3), 0.67-2.05 (15H, 1+4+5+6+8+9+11).

^{1}H-NMR (400 MHz, $CDCl_3$) of B-MG-BPMA-L 15 mol%: δ [ppm] = 7.63 (2H, 15), 7.52 (2H, 17), 6.58 (1H, 14), 6.24 (2H, 16), 4.19-4.82 (3H, 2+10), 3.67 (2H, 13), 2.87-3.46 (2H, 7), 2.66 (2H, 12), 2.09-2.61 (2H, 3), 0.64-2.04 (15H, 1+4+5+6+8+9+11).

^{1}H-NMR (400 MHz, $CDCl_3$) of SB-MG-BPMA-L 5 mol%: δ [ppm] = 7.66 (2H, 15), 7.52 (2H, 17), 6.24 (2H, 16), 4.15-4.77 (3H, 2+10), 3.72 (2H, 13), 2.86-3.53 (2H, 7), 2.12-2.64 (2H, 3), 0.65-2.05 (15H, 1+4+5+6+8+9+11).

^{1}H-NMR (400 MHz, $CDCl_3$) of SB-MG-BPMA-L 10 mol%: δ [ppm] = 7.64 (2H, 15), 7.52 (2H, 17), 6.62 (1H, 14), 6.24 (2H, 16), 4.17-4.80 (3H, 2+10), 3.68 (2H, 13), 2.86-3.50 (2H, 7), 2.68 (2H, 12), 2.10-2.60 (2H, 3), 0.61-2.04 (15H, 1+4+5+6+8+9+11).

^{1}H-NMR (400 MHz, $CDCl_3$) of SB-MG-BPMA-L 15 mol%: δ [ppm] = 7.64 (2H, 15), 7.52 (2H, 17), 6.58 (1H, 14), 6.23 (2H, 16), 4.19-4.80 (3H, 2+10), 3.67 (2H, 13), 2.84-3.46 (2H, 7), 2.67 (2H, 12), 2.11-2.61 (2H, 3), 0.55-2.05 (15H, 1+4+5+6+8+9+11).

Table S13. Amounts of used VCL and BPMA-L for microgel synthesis and the corresponding gravimetrically determined yields.

sample	m(VCL) [g]	m(BPMA-L) [g]	yield [%]
B-MG-BPMA-L 5 mol%	1.257	0.145	92
B-MG-BPMA-L 10 mol%	1.191	0.288	87
B-MG-BPMA-L 15 mol%	1.124	0.432	85
SB-MG-BPMA-L 5 mol%	1.257	0.144	95
SB-MG-BPMA-L 10 mol%	1.190	0.288	84
SB-MG-BPMA-L 15 mol%	1.124	0.433	83

Table S14. Theoretical BPMA-L content and determined BPMA-L content of microgels and copolymer *via* ^{1}H-NMR and Raman spectroscopy.

**sample**	**BPMA-L$_{theor}$ [mol%]**	**BPMA-L$_{NMR}$ [mol%]**	**BPMA-L$_{Raman}$ [mol%]**
B-MG-BPMA-L 5 mol%	5.0	3.7	4.2
B-MG-BPMA-L 10 mol%	10.0	9.1	9.1
B-MG-BPMA-L 15 mol%	15.0	17.1	12.6
SB-MG-BPMA-L 5 mol%	5.0	5.1	4.1
SB-MG-BPMA-L 10 mol%	10.0	12.6	9.1
SB-MG-BPMA-L 15 mol%	15.0	21.6	14.0
P(VCL)$_{0.85}$-(BPMA-L)$_{0.15}$	15.0	18.8	12.2

Table S15. Hydrodynamic radii and PDI values of BPMA-L microgels at 20 °C and 50 °C determined *via* DLS.

**sample**	**R_h 20 °C [nm]**	**PDI 20 °C**	**R_h 50 °C [nm]**	**PDI 50 °C**
B-MG-BPMA-L 5 mol%	208.9 ± 3.7	0.131	125.4 ± 3.6	0.132
B-MG-BPMA-L 10 mol%	158.5 ± 9.4	0.100	118.5 ± 1.3	0.041
B-MG-BPMA-L 15 mol%	173.0 ± 9.0	0.057	136.9 ± 0.9	0.014
SB-MG-BPMA-L 5 mol%	287.4 ± 6.8	0.104	148.3 ± 2.5	0.003
SB-MG-BPMA-L 10 mol%	199.8 ± 9.3	0.038	132.03 ± 2.3	0.091
SB-MG-BPMA-L 15 mol%	172.3 ± 9.8	0.050	137.9 ± 3.3	0.040

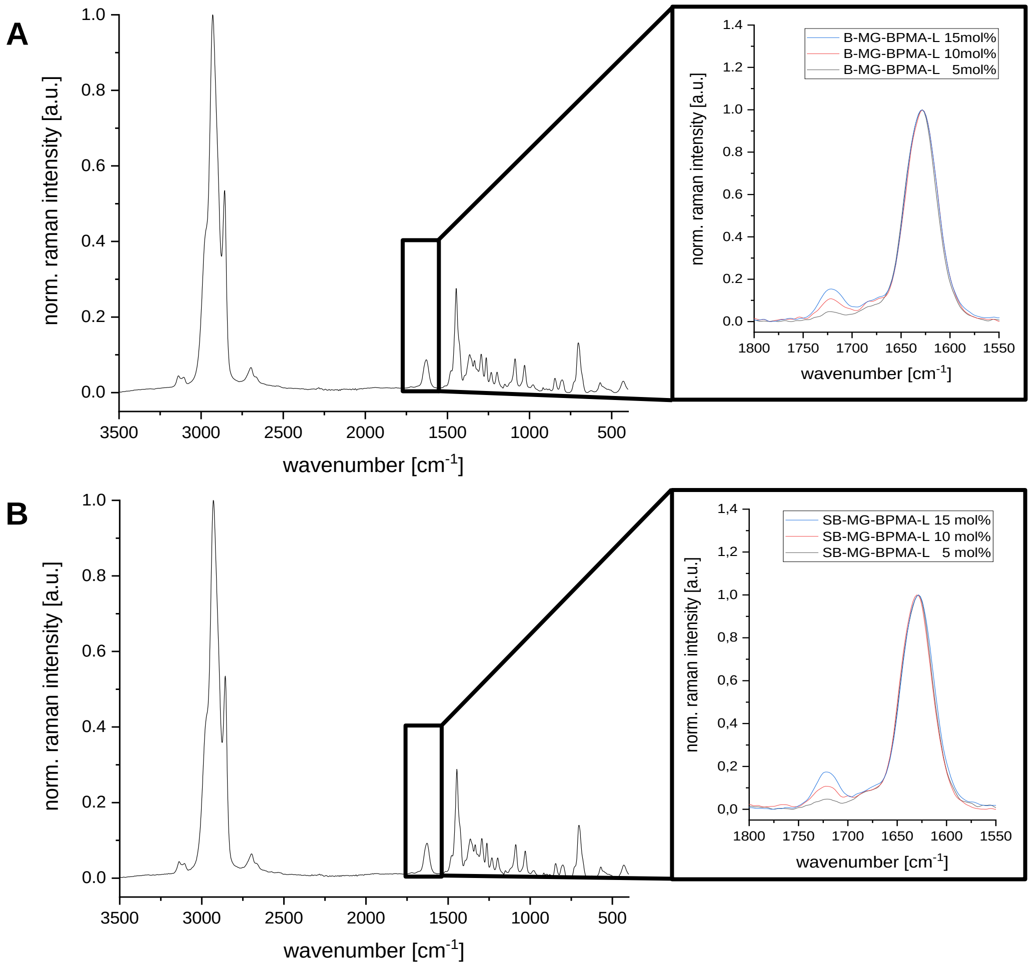

Figure S4. Raman spectra of the B-MG-BPMA-L 5 mol% (A) and SB-MG-BPMA-L 5 mol% (B) microgels with enlarged view on the wavenumber range between 1550 cm^{-1} to 1800 cm^{-1} of all respective microgels.

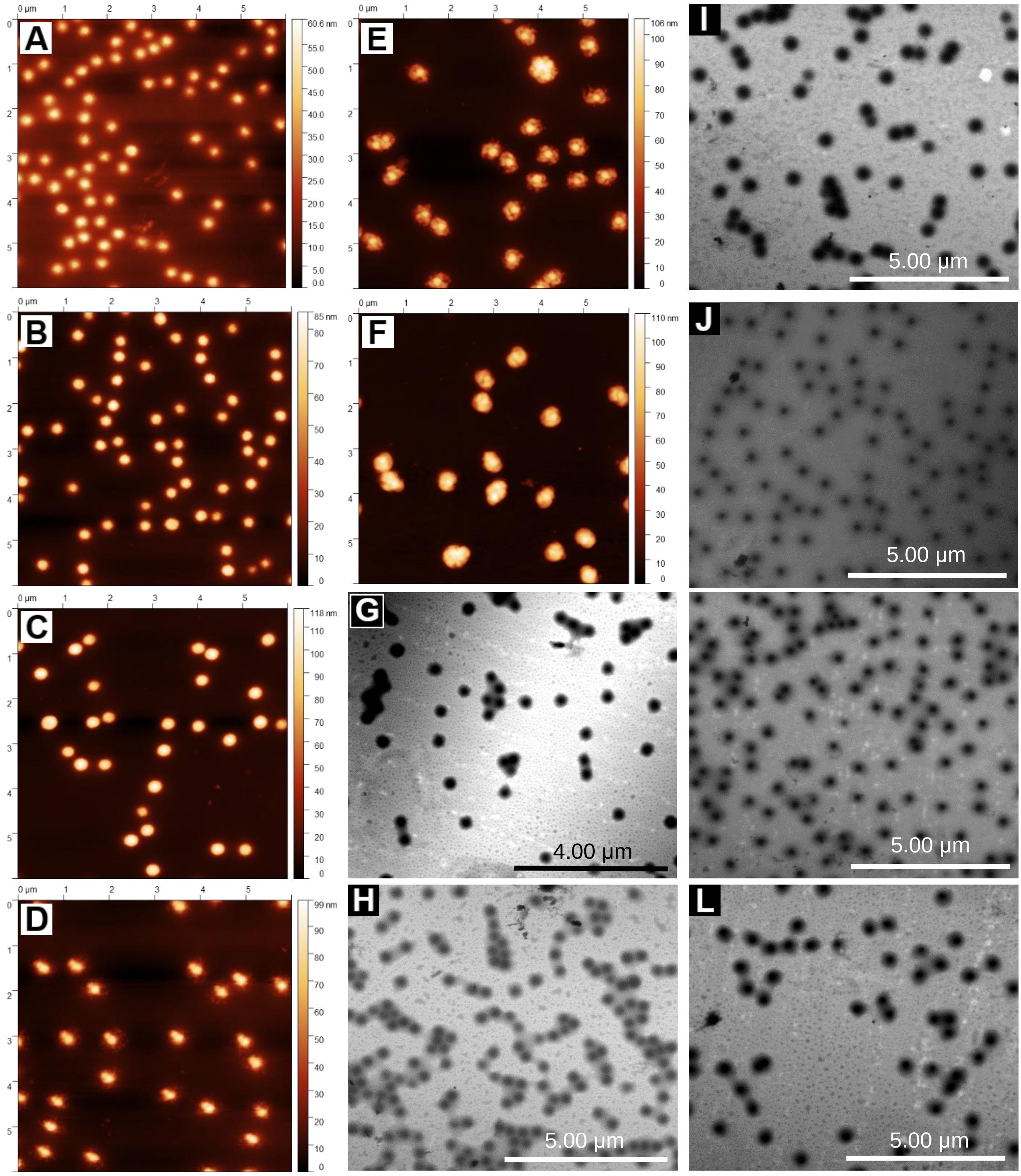

Figure S5. AFM (A-F) and STEM (G-L) images of the BPMA-L microgels. The batch microgels with 5 mol% (A & G), 10 mol% (B & H) and 15 mol% (C & I) are ordered by increasing content of BPMA-L for the respective microscopy method. The same applies for the semi-batch microgels with 5 mol% (D & J), 10 mol% (E & K) and 15 mol% (F & L).

Table S16. Diameter of the B-MG- and SB-MG-BPMA-L microgels determined *via* STEM images.

sample	diameter [nm]
B-MG-BPMA-L 5 mol%	354.7 ± 11.0
B-MG-BPMA-L 10 mol%	445.2 ± 23.1
B-MG-BPMA-L 15 mol%	506.0 ± 27.9
SB-MG-BPMA-L 5 mol%	413.8 ± 21.1
SB-MG-BPMA-L 10 mol%	422.3 ± 32.9
SB-MG-BPMA-L 15 mol%	536.8 ± 34.7

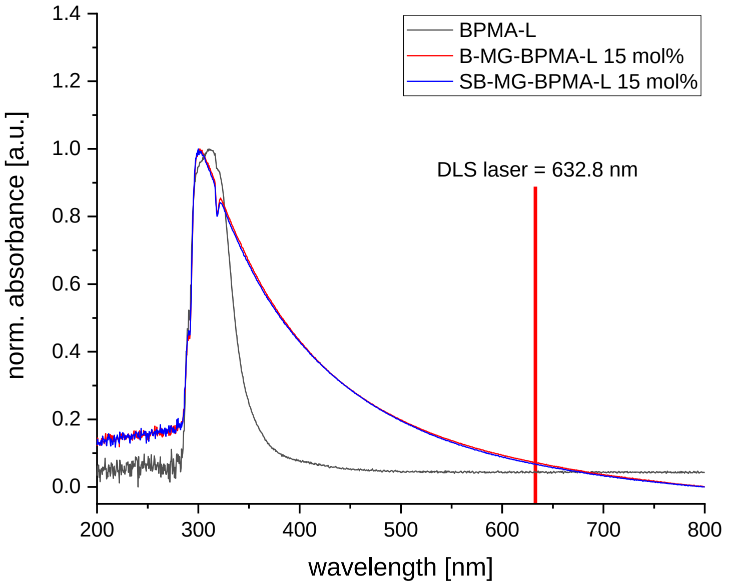

Figure S6. UV/Vis spectra of BPMA-L, B-MG-BPMA-L 15 mol%, and SB-MG-BPMA-L 15 mol% with indication of the wavelength used by the DLS laser.

Synthesis of BPMA-LP Microgels

The core-shell microgels were prepared in a similar manner to the PMA-S microgels, proceeding from VCL (amounts see **Table S17)**, BIS (0.285 mmol, 3 mol%), BPMA-LP

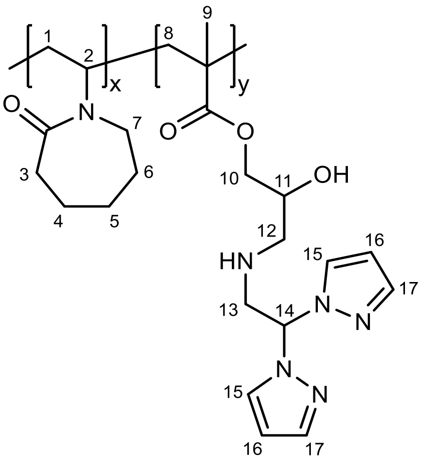

(amounts see **Table S17**) and AMPA (0.076 mmol, 0.8 mol%).

^{1}H-NMR (400 MHz, $CDCl_3$) of B-MG-BPMA-LP 5 mol%: δ [ppm] = 7.69 (2H, 15), 7.53 (2H, 17), 6.24 (2H, 16), 4.08-4.83 (3H, 2+10), 2.84-3.62 (3H, 7+11), 2.13-2.79 (2H, 3), 0.61-2.11 (13H, 1+4+5+6+8+9).

^{1}H-NMR (400 MHz, $CDCl_3$) of B-MG-BPMA-LP 10 mol%: δ [ppm] = 7.67 (2H, 15), 7.52 (2H, 17), 6.78 (1H, 14), 6.24 (2H, 16), 4.18-4.85 (3H, 2+10), 3.79 (2H, 13), 2.87-3.47 (3H, 7+11), 2.76 (2H, 12), 2.15-2.65 (2H, 3), 0.63-2.10 (13H, 1+4+5+6+8+9).

^{1}H-NMR (400 MHz, $CDCl_3$) of B-MG-BPMA-LP 15 mol%: δ [ppm] = 7.65 (2H, 15), 7.51 (2H, 17), 6.70 (1H, 14), 6.23 (2H, 16), 4.15-4.92 (3H, 2+10), 3.76 (2H, 13), 2.86-3.50 (3H, 7+11), 2.70 (2H, 12), 2.13-2.64 (2H, 3), 0.61-2.11 (13H, 1+4+5+6+8+9).

^{1}H-NMR (400 MHz, $CDCl_3$) of SB-MG-BPMA-LP 5 mol%: δ [ppm] = 7.71 (2H, 15), 7.54 (2H, 17), 6.86 (1H, 14), 6.25 (2H, 16), 4.08-4.79 (3H, 2+10), 2.87-3.55 (3H, 7+11), 2.15-2.78 (2H, 3), 0.62-2.09 (13H, 1+4+5+6+8+9).

^{1}H-NMR (400 MHz, $CDCl_3$) of SB-MG-BPMA-LP 10 mol%: δ [ppm] = 7.67 (2H, 15), 7.51 (2H, 17), 6.81 (1H, 14), 6.23 (2H, 16), 4.19-4.82 (3H, 2+10), 3.80 (2H, 13), 2.85-3.46 (3H, 7+11), 2.77 (2H, 12), 2.12-2.64 (2H, 3), 0.64-2.05 (13H, 1+4+5+6+8+9).

^{1}H-NMR (400 MHz, $CDCl_3$) of SB-MG-BPMA-LP 15 mol%: δ [ppm] = 7.65 (2H, 15), 7.51 (2H, 17), 6.73 (1H, 14), 6.23 (2H, 16), 4.20-4.88 (3H, 2+10), 3.78 (2H, 13), 2.88-3.49 (3H, 7+11), 2.74 (2H, 12), 2.16-2.64 (2H, 3), 0.73-2.09 (13H, 1+4+5+6+8+9).

Table S17. Amounts of used VCL and BPMA-LP for microgel synthesis and the corresponding gravimetrically determined yields.

sample	m(VCL) [g]	m(BPMA-LP) [g]	yield [%]
B-MG-BPMA-LP 5 mol%	1.257	0.152	86
B-MG-BPMA-LP 10 mol%	1.191	0.305	53
B-MG-BPMA-LP 15 mol%	1.125	0.458	70
SB-MG-BPMA-LP 5 mol%	1.257	0.152	85
SB-MG-BPMA-LP 10 mol%	1.190	0.311	56
SB-MG-BPMA-LP 15 mol%	1.125	0.458	70

Table S18. Hydrodynamic radii and PDI values of BPMA-LP microgels at 20 °C and 50 °C determined *via* DLS.

sample	R_h 20 °C [nm]	PDI 20 °C	R_h 50 °C [nm]	PDI 50 °C
B-MG-BPMA-LP 5 mol%	206.5 ± 2.8	0.076	165.0 ± 6.5	0.127
B-MG-BPMA-LP 10 mol%	118.0 ± 1.7	0.046	175.5 ± 4.3	0.421
B-MG-BPMA-LP 15 mol%	144.0 ± 0.9	0.078	168.5 ± 7.8	0.150
SB-MG-BPMA-LP 5 mol%	272.0 ± 18.4	0.231	253.5 ± 2.5	0.090
SB-MG-BPMA-LP 10 mol%	201.5 ± 5.9	0.040	160.5 ± 6.2	0.154
SB-MG-BPMA-LP 15 mol%	170.5 ± 4.1	0.030	163.0 ± 2.3	0.064

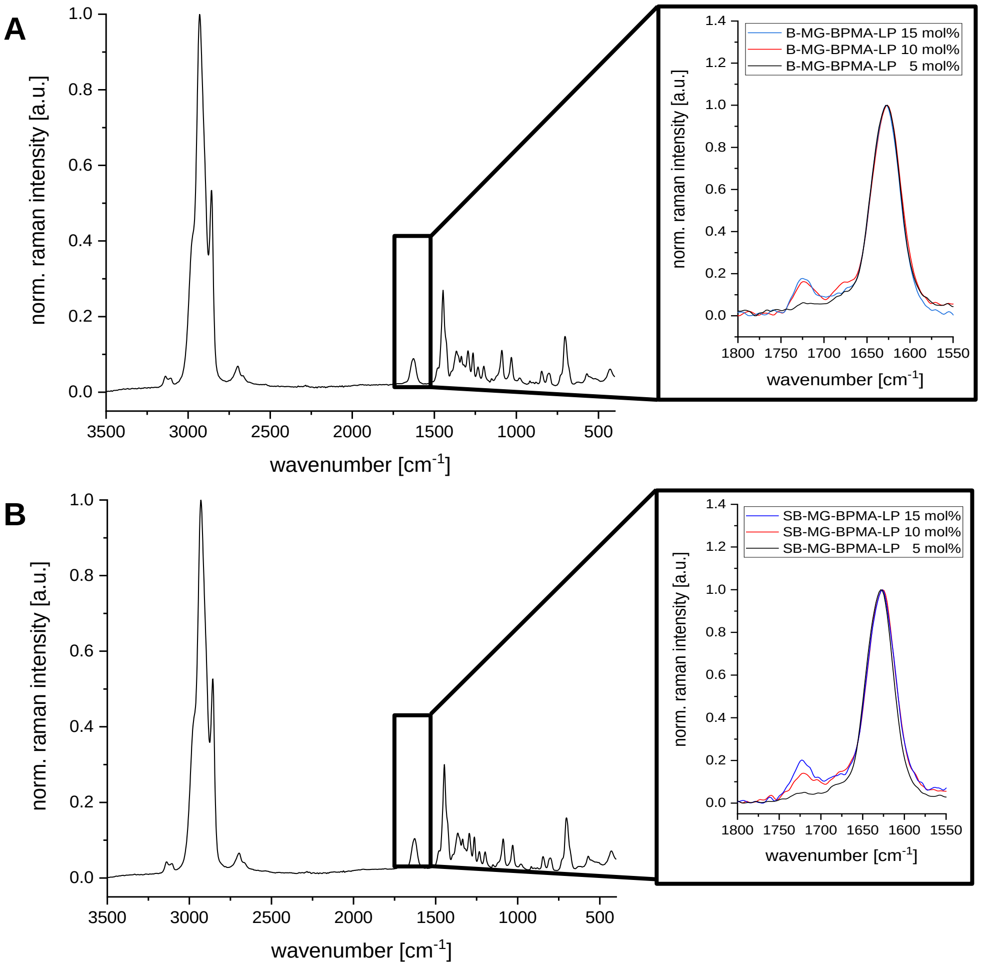

Figure S7. Raman spectra of the B-MG-BPMA-LP 5 mol% (A) and SB-MG-BPMA-LP 5 mol% (B) microgels with enlarged view on the wavenumber range between 1550 cm^{-1} to 1800 cm^{-1} of all respective microgels.

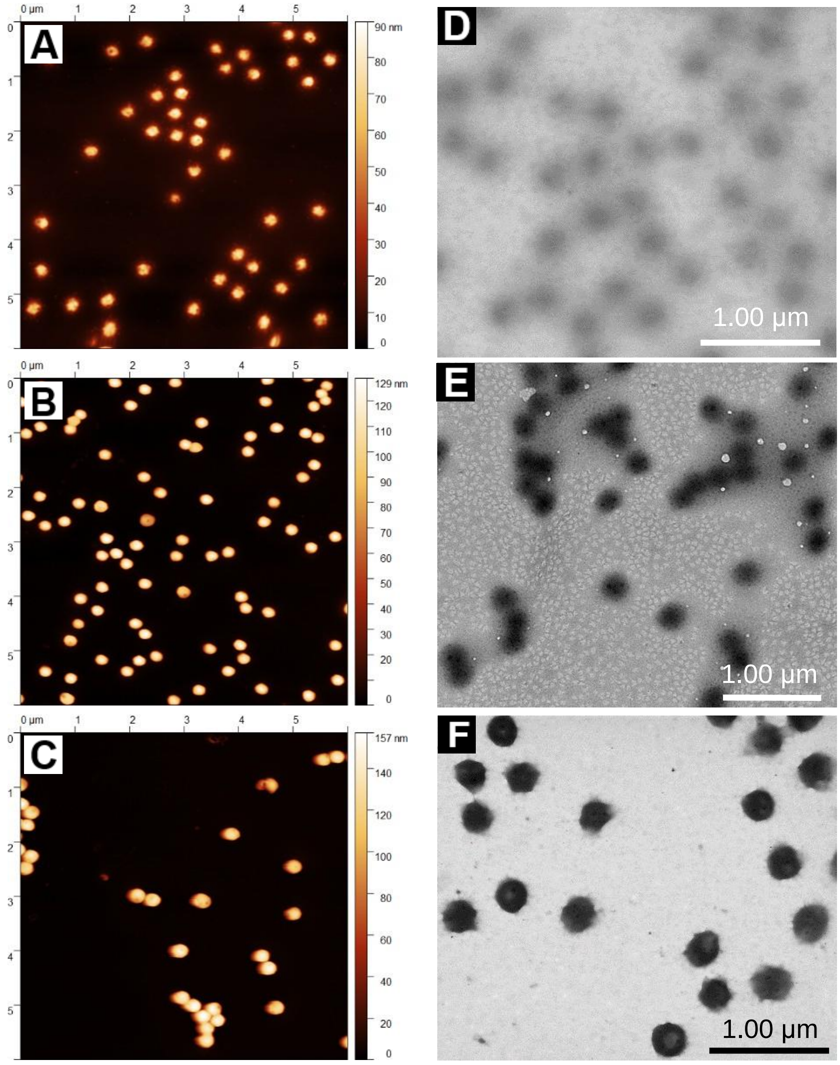

Figure S8. AFM (A-C) and STEM/TEM (D-F) images of the B-MG-BPMA-LP microgels ordered by increase of BPMA-LP content. On the top are the microgels with 5 mol% (A & D), at the middle with 10 mol% (B & E) and at the bottom with 15 mol% (C & F).

Table S19. Diameter, length, and width of the B-MG- and SB-MG-BPMA-LP microgels determined *via* STEM/TEM images.

sample	diameter [nm]	length [nm]	width [nm]
B-MG-BPMA-LP 5 mol%	257.5 ± 10.2	-	-
B-MG-BPMA-LP 10 mol%	235.8 ± 16.6	-	-
B-MG-BPMA-LP 15 mol%	261.7 ± 18.9	-	-
SB-MG-BPMA-LP 5 mol%	462.7 ± 56.7	-	-
SB-MG-BPMA-LP 10 mol%	293.8 ± 26.8	-	-
SB-MG-BPMA-LP 15 mol%	-	313.4 ± 22.2	193.3 ± 21.3

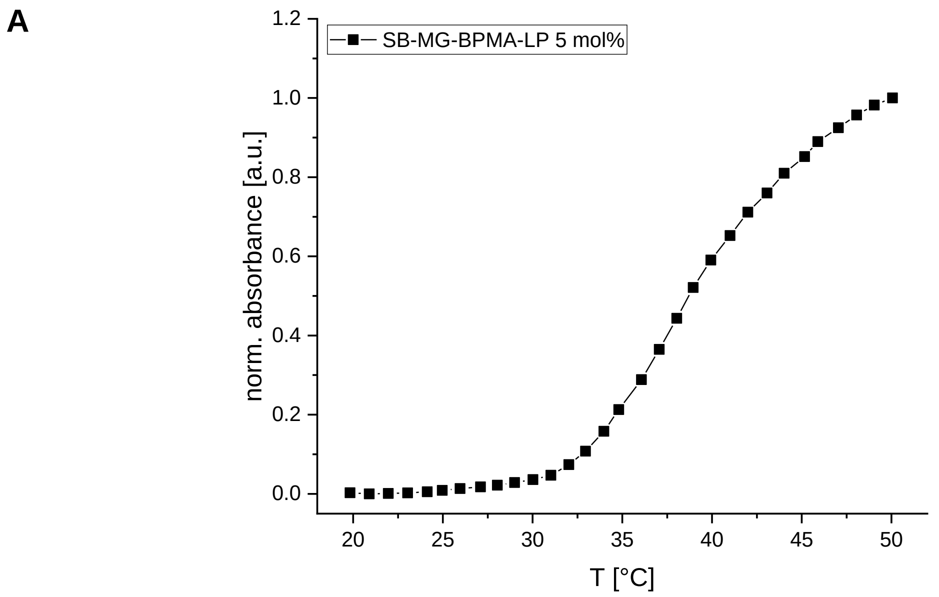

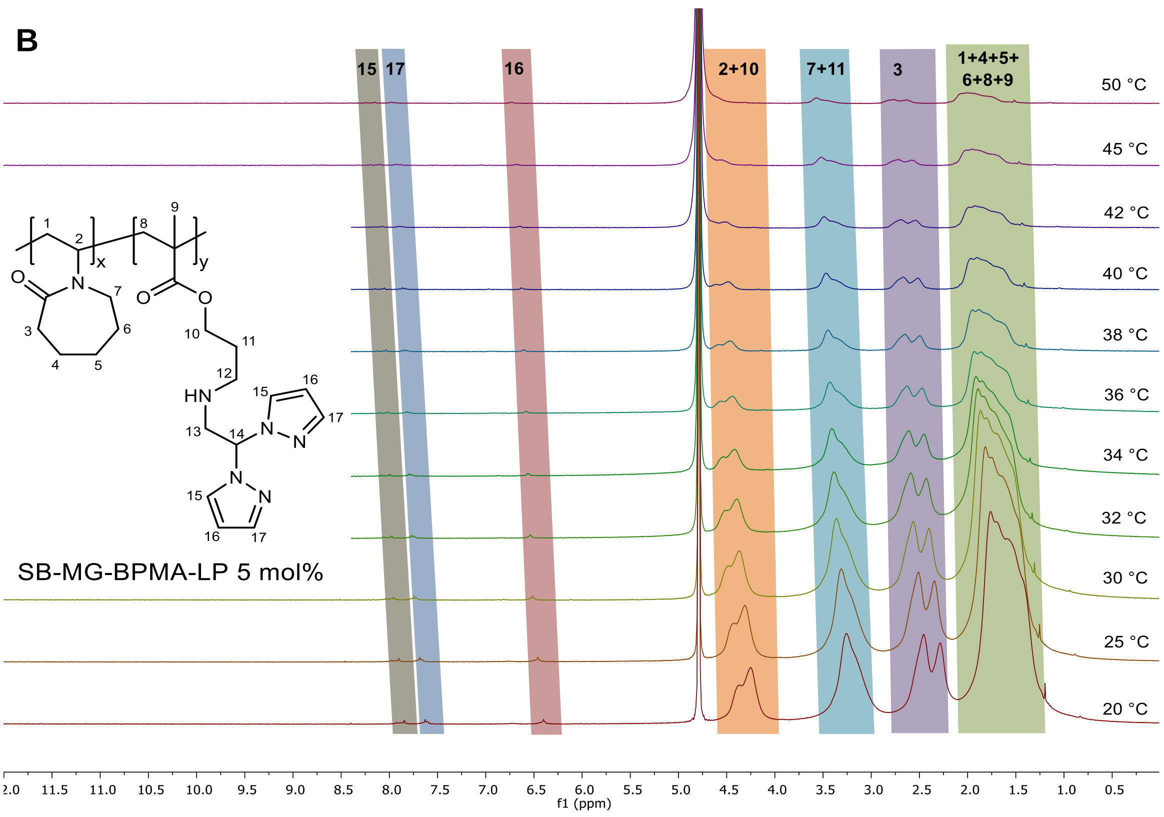

Figure S9. Cloud point study of SB-MG-BPMA-LP 5 mol% microgels *via* UV/Vis spectroscopy (A). ^{1}H-NMR spectra of SB-MG-BPMA-LP 5 mol% microgels measured between 20 and 50 °C in D_2O (B).

Synthesis of $P(VCL)_{0.85}$-$(PMA\text{-}S)_{0.15}$ for the identification of π-π-stacking

The copolymer $P(VCL)_{0.85}$-$(PMA\text{-}S)_{0.15}$ was synthesized in an analogous way to the described microgel synthesis with the absence of crosslinker using VCL (6.375 mmol, 85 mol%), PMA-S (1.125 mmol, 15 mol%) and AMPA (0.060 mmol, 0.8 mol%). The obtained copolymer was dialyzed against deionized water (MWCO: 6.000-8.000 Da) for 5 days and lyophilized (0.301 g, 28 %).

^{1}H-NMR (400 MHz, $CDCl_3$) with c = 30 mg/mL: δ [ppm] = 7.58 (1H, 14), 7.47 (1H, 12), 6.20 (1H, 13), 4.55 (1H, 2), 4.26 (2H, 11), 3.51-3.87 (2H, 10), 2.82-3.39 (2H, 7), 2.64 (1H, 15), 2.12-2.77 (2H, 3), 0.68-2.07 (13H, 1+4+5+6+8+9).

^{1}H-NMR (400 MHz, $CDCl_3$) with c = 90 mg/mL: δ [ppm] = 7.57 (1H, 14), 7.46 (1H, 12), 6.18 (1H, 13), 4.54 (1H, 2), 4.24 (2H, 11), 3.45-3.89 (2H, 10), 3.18 (1H, 15), 2.73-3.43 (2H, 7), 2.09-2.68 (2H, 3), 0.66-2.06 (13H, 1+4+5+6+8+9).

^{1}H-NMR (400 MHz, $CDCl_3$) with c = 150 mg/mL: δ [ppm] = 7.54 (1H, 14), 7.43 (1H, 12), 6.16 (1H, 13), 4.50 (1H, 2), 4.23 (2H, 11), 3.41 (1H, 15), 2.74-3.93 (4H, 7+10), 2.07-2.67 (2H, 3), 0.59-2.05 (13H, 1+4+5+6+8+9).

Synthesis of $P(VCL)_{0.85}$-$(BPMA\text{-}S)_{0.15}$ for the identification of π-π-stacking

The copolymer $P(VCL)_{0.85}$-$(BPMA\text{-}S)_{0.15}$ was synthesized in an analogous way to the described microgel synthesis with the absence of crosslinker using VCL (6.375 mmol, 85 mol%), BPMA-S (1.125 mmol, 15 mol%) and AMPA (0.060 mmol, 0.8 mol%). The obtained copolymer was dialyzed against deionized water (MWCO: 3.500 Da) for 5 days and lyophilized (0.956 g, 82 %).

^{1}H-NMR (400 MHz, $CDCl_3$) with c = 30 mg/mL: δ [ppm] = 7.73 (2H, 12), 7.51 (2H, 14), 6.75 (1H, 11), 6.23 (2H, 13), 3.98-4.71 (3H, 2+10), 2.83-3.48 (2H, 7), 2.15-2.76 (2H, 3), 2.56 (1H, 15), 0.51-2.10 (13H, 1+4+5+6+8+9).

^{1}H-NMR (400 MHz, $CDCl_3$) with c = 90 mg/mL: δ [ppm] = 7.71 (2H, 12), 7.49 (2H, 14), 6.71 (1H, 11), 6.20 (2H, 13), 3.86-5.14 (3H, 2+10), 2.72-3.60 (2H, 7), 3.07 (1H, 15), 2.10-2.68 (2H, 3), 0.38-2.07 (13H, 1+4+5+6+8+9).

^{1}H-NMR (400 MHz, $CDCl_3$) with c = 150 mg/mL: δ [ppm] = 7.69 (2H, 12), 7.48 (2H, 14), 6.70 (1H, 11), 6.18 (2H, 13), 3.88-5,01 (3H, 2+10), 4.72 (1H, 15), 2.74-3.65 (2H, 7), 2.09-2.69 (2H, 3), 0.38-2.05 (13H, 1+4+5+6+8+9).

Synthesis of $P(VCL)_{0.85}$-$(TPMA\text{-}S)_{0.15}$ for the identification of *π*-*π*-stacking

The copolymer $P(VCL)_{0.85}$-$(TPMA\text{-}S)_{0.15}$ was synthesized in an analogous way to the described microgel synthesis with the absence of crosslinker using VCL (6.375 mmol, 85 mol%), TPMA-S (1.125 mmol, 15 mol%) and AMPA (0.060 mmol, 0.8 mol%). The obtained copolymer was dialyzed against deionized water (MWCO: 3.500 Da) for 5 days and lyophilized (0.510 g, 41 %).

^{1}H-NMR (400 MHz, $CDCl_3$) with c = 30 mg/mL: δ [ppm] = 7.65 (3H, 13), 6.99 (3H, 11), 6.30 (3H, 12), 4.81-5.37 (2H, 10), 4.16-4.68 (1H, 2), 2.82-3.50 (2H, 7), 2.13-2.73 (2H, 3), 0.52-2.05 (13H, 1+4+5+6+8+9).

^{1}H-NMR (400 MHz, $CDCl_3$) with c = 90 mg/mL: δ [ppm] = 7.63 (3H, 13), 6.95 (3H, 11), 6.28 (3H, 12), 4.82-5.37 (2H, 10), 4.06-4.64 (1H, 2), 2.72-3.48 (2H, 7), 2.09-2.65 (2H, 3), 0.44-2.06 (13H, 1+4+5+6+8+9).

^{1}H-NMR (400 MHz, $CDCl_3$) with c = 150 mg/mL: δ [ppm] = 7.61 (3H, 13), 6.93 (3H, 11), 6.26 (3H, 12), 4.81-5.34 (2H, 10), 4.06-4.65 (1H, 2), 2.73-3.56 (2H, 7), 2.07-2.68 (2H, 3), 0.42-2.04 (13H, 1+4+5+6+8+9).

Synthesis of $P(VCL)_{0.85}$-$(BPMA\text{-}L)_{0.15}$ for the identification of *π*-*π*-stacking

The copolymer $P(VCL)_{0.85}$-$(BPMA\text{-}L)_{0.15}$ was synthesized in an analogous way to the described microgel synthesis with the absence of crosslinker using VCL (6.375 mmol,

85 mol%), BPMA-L (1.125 mmol, 15 mol%) and AMPA (0.060 mmol, 0.8 mol%). The obtained copolymer was dialyzed against deionized water (MWCO: 3.500 Da) for 5 days and lyophilized (1.032 g, 84 %).

^{1}H-NMR (400 MHz, $CDCl_3$) with c = 30 mg/mL: δ [ppm] = 7.63 (2H, 15), 7.51 (2H, 17), 6.58 (1H, 14), 6.23 (2H, 16), 4.18-4.83 (3H, 2+10), 3.68 (2H, 13), 2.86-3.50 (2H, 7), 2.68 (2H, 12), 2.12-2.61 (2H, 3), 0.56-2.12 (15H, 1+4+5+6+8+9+11).

^{1}H-NMR (400 MHz, $CDCl_3$) with c = 90 mg/mL: δ [ppm] = 7.59 (2H, 15), 7.48 (2H, 17), 6.52 (1H, 14), 6.20 (2H, 16), 4.13-4.84 (3H, 2+10), 3.63 (2H, 13), 2.84-3.47 (2H, 7), 2.63 (2H, 12), 2.11-2.58 (2H, 3), 0.57-2.08 (15H, 1+4+5+6+8+9+11).

^{1}H-NMR (400 MHz, $CDCl_3$) with c = 150 mg/mL: δ [ppm] = 7.58 (2H, 15), 7.46 (2H, 17), 6.51 (1H, 14), 6.18 (2H, 16), 4.12-4.80 (3H, 2+10), 3.61 (2H, 13), 2.75-3.44 (2H, 7), 2.62 (2H, 12), 2.11-2.55 (2H, 3), 0.56-2.04 (15H, 1+4+5+6+8+9+11).

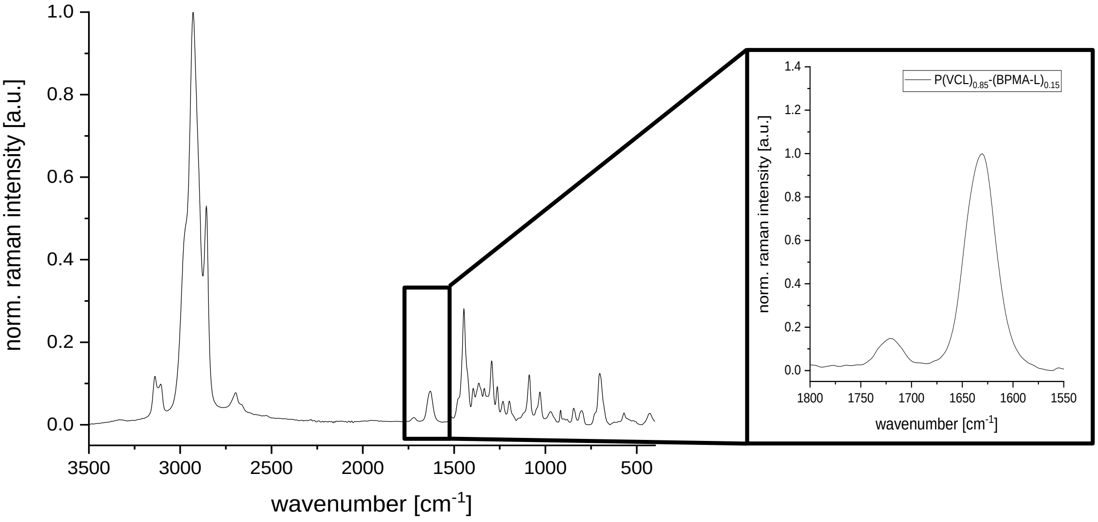

Figure S10. Raman spectrum of the copolymer $P(VCL)_{0.85}$-$(BPMA\text{-}L)_{0.15}$ with enlarged view on the wavenumber range between 1550 cm^{-1} to 1800 cm^{-1}.

Synthesis of $P(VCL)_{0.85}$-$(BPMA\text{-}LP)_{0.15}$ for the identification of π-π-stacking

The copolymer $P(VCL)_{0.85}$-$(BPMA\text{-}LP)_{0.15}$ was synthesized in an analogous way to the described microgel synthesis with the absence of crosslinker using VCL (6.205 mmol,

85 mol%), BPMA-LP (1.095 mmol, 15 mol%) and AMPA (0.058 mmol, 0.8 mol%). The obtained copolymer was dialyzed against deionized water (MWCO: 12.000-14.000 Da) for 5 days and lyophilized (0.357 g, 29 %).

^{1}H-NMR (400 MHz, $CDCl_3$) with c = 30 mg/mL: δ [ppm] = 7.63 (2H, 15), 7.52 (2H, 17), 6.56 (1H, 14), 6.28 (2H, 16), 4.83-4.19 (3H, 2+10), 3.69 (2H, 13), 3.48-2.87 (3H, 7+11), 2.67 (2H, 12), 2.61-2.05 (2H, 3), 2.02-0.76 (11H, 1+4+5+6+8+9).

^{1}H-NMR (400 MHz, $CDCl_3$) with c = 90 mg/mL: δ [ppm] = 7.61 (2H, 15), 7.49 (2H, 17), 6.55 (1H, 14), 6.21 (2H, 16), 4.79-4.19 (3H, 2+10), 3.67 (2H, 13), 3.49-2.90 (3H, 7+11), 2.66 (2H, 12), 2.58-2.11 (2H, 3), 2.07-0.73 (11H, 1+4+5+6+8+9).

^{1}H-NMR (400 MHz, $CDCl_3$) with c = 150 mg/mL: δ [ppm] = 7.58 (2H, 15), 7.46 (2H, 17), 6.51 (1H, 14), 6.17 (2H, 16), 5.02-3.96 (3H, 2+10), 3.64 (2H, 13), 3.45-2.74 (3H, 7+11), 2.61 (2H, 12), 2.55-2.07 (2H, 3), 2.05-0.65 (11H, 1+4+5+6+8+9).

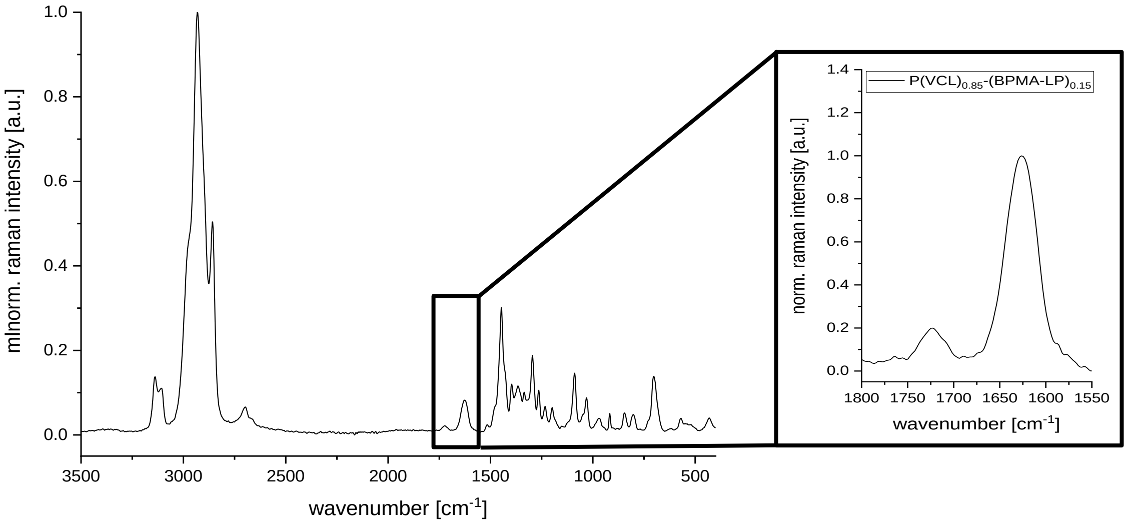

Figure S11. Raman spectrum of the copolymer $P(VCL)_{0.85}$-$(BPMA\text{-}LP)_{0.15}$ with enlarged view on the wavenumber range between 1550 cm^{-1} to 1800 cm^{-1}.

Computer Simulations

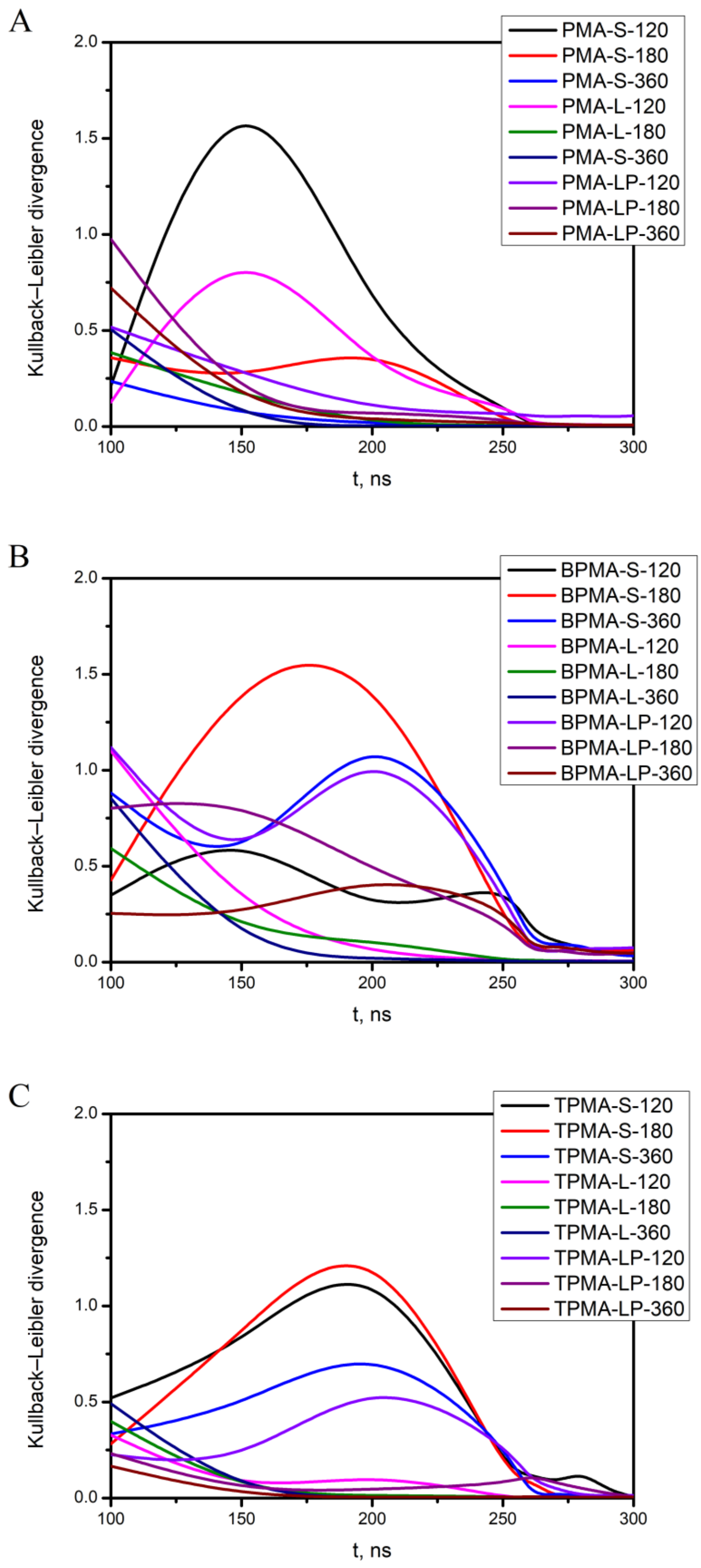

Figure S12. The Kullback–Leibler divergence for PMA (A), BPMA (B), and TPMA (C) systems during the simulation time.

The radial distribution function (RDF) for pyrazole groups was calculated to analyze their pair formation (**Figure S13**). The presence of two peaks in RDF indicates triplet formation. Such behavior appears in all TPMA monomers. The start of peak splitting can be seen in BPMA-LP and PMA-S systems. The other system formed only doublets.

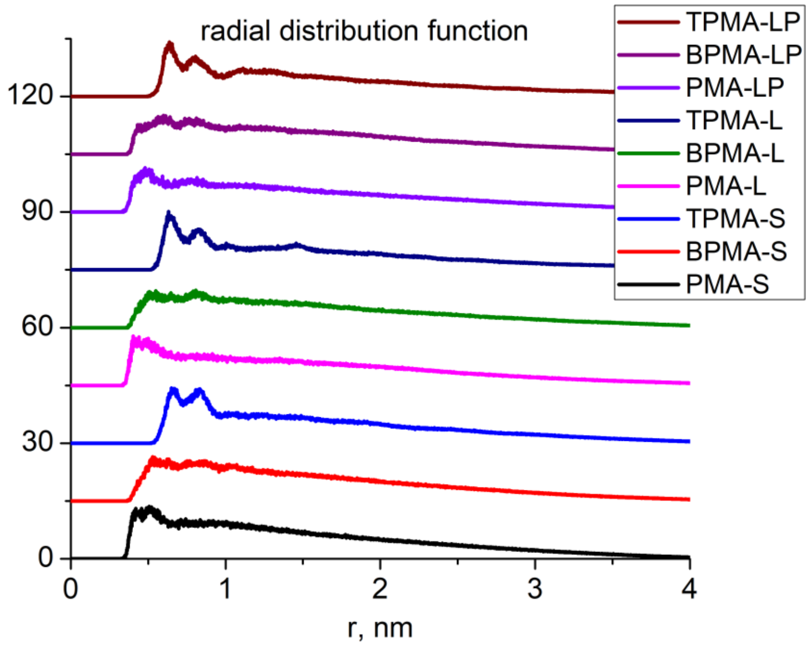

Figure S13. The radial distribution function (RDF) for pyrazole groups in the solvent system. All plots are on the same length scale and shifted by 15 correspondingly.

In order to understand the initial stages of the microgel synthesis, we run a series of surface experiments. The hypothesis was to mimic a VCL microgel surface. Since the x- and y- box sizes are 13.5 nm and 12.1 nm, respectively, the curved microgel surface can be approximated by the flat layer of VCL oligomers. There are two types of oligomers consisting of 5 and 10 VCL monomers, which were set regularly in hexagonal order at the constant distance of 0.9 nm. One end of oligomers was fixed in space. Due to the two-thirds of VCL monomers that had reacted when BPMA residues were added we added free VLC monomers to the simulation box with the corresponding concentration.

The pyrazole monomers were solvated in a water/methanol mixture (2.2 vol%) in the box for three different concentrations. The overall composition for solvent and surface simulations is presented in **Table S20**. The snapshots of the surface system's final state are presented in **Figure S14**. We calculate density profiles in **Figure S15** that show a distribution of the components along the normal to the surface.

**sample**	**pyrazole monomers**	**methanol molecules**	**water molecules**	**VCL surface oligomers**	**VCL free monomers**
solvent case	100	500	~20500	-	100
surface case	120/180/360	1366	~55000	100	85

Table S20. The content of simulation systems.

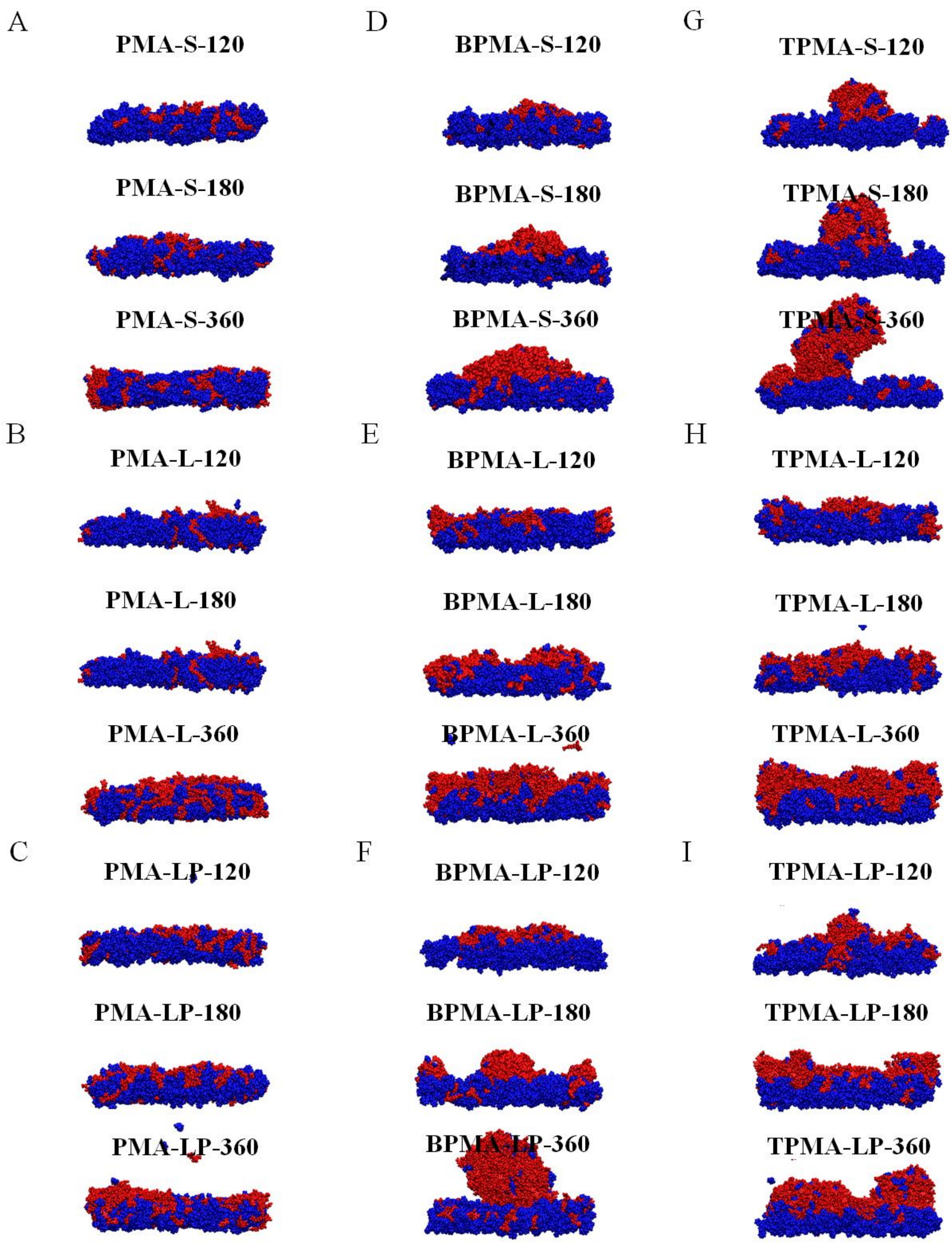

Figure S14. Final states of surface systems with different concentrations and types (A-I) of monomers are present. Surface oligomers and VCL monomers are shown in blue and pyrazole monomers are in red. Water and methanol molecules are not shown.

Aromatic monomers with one pyrazole residue wet the surface regardless of a spacer type completely (**Figure S14** and **Figure S15 A-C**). They incorporate into the inner structure of the surface between oligomers. This complete wetting behavior divides into two regimes. The

first regime is then pyrazole monomers repeat the surface density profile and all monomers introduced in the surface media. Among such systems are PMA-S and PMA-L. Another state is typical for PMA-LP, BPMA-L, TPMA-L, and TPMA-LP. Here, the density profiles of pyrazole monomers tend to a surface boundary at low concentrations (N=120) and make a flat layer at high concentrations. In the last case, the density curve has the same shape but shifted by 1 nm from the surface center. The next type of behavior is partial wetting which we can see only in the BPMA-S system. BPMA-S monomers form a denser aggregate on the VCL surface with increasing the concentration. The last behavior type is a bad wetting of the oligomer surface by pyrazole monomers in BPMA-LP and TPMA-S systems. Monomers form an aggregate that incorporates into the water phase at a high concentration (N=360) and the density profile spreads from 1 to 9 nm with no clear peaks and the TPMA-S system stays under bad wetting conditions. On the other hand, we can see that the BPMA-LP system follows from the complete wetting at N=120 to the partial wetting at N=180 and the bad wetting at N=360, thus, all types of monomers except BPMA-LP show only one type of behavior.

Free VCL monomers locate in the pyrazole monomer phase mostly. That can be seen as small blue segments in the red pyrazole phase.

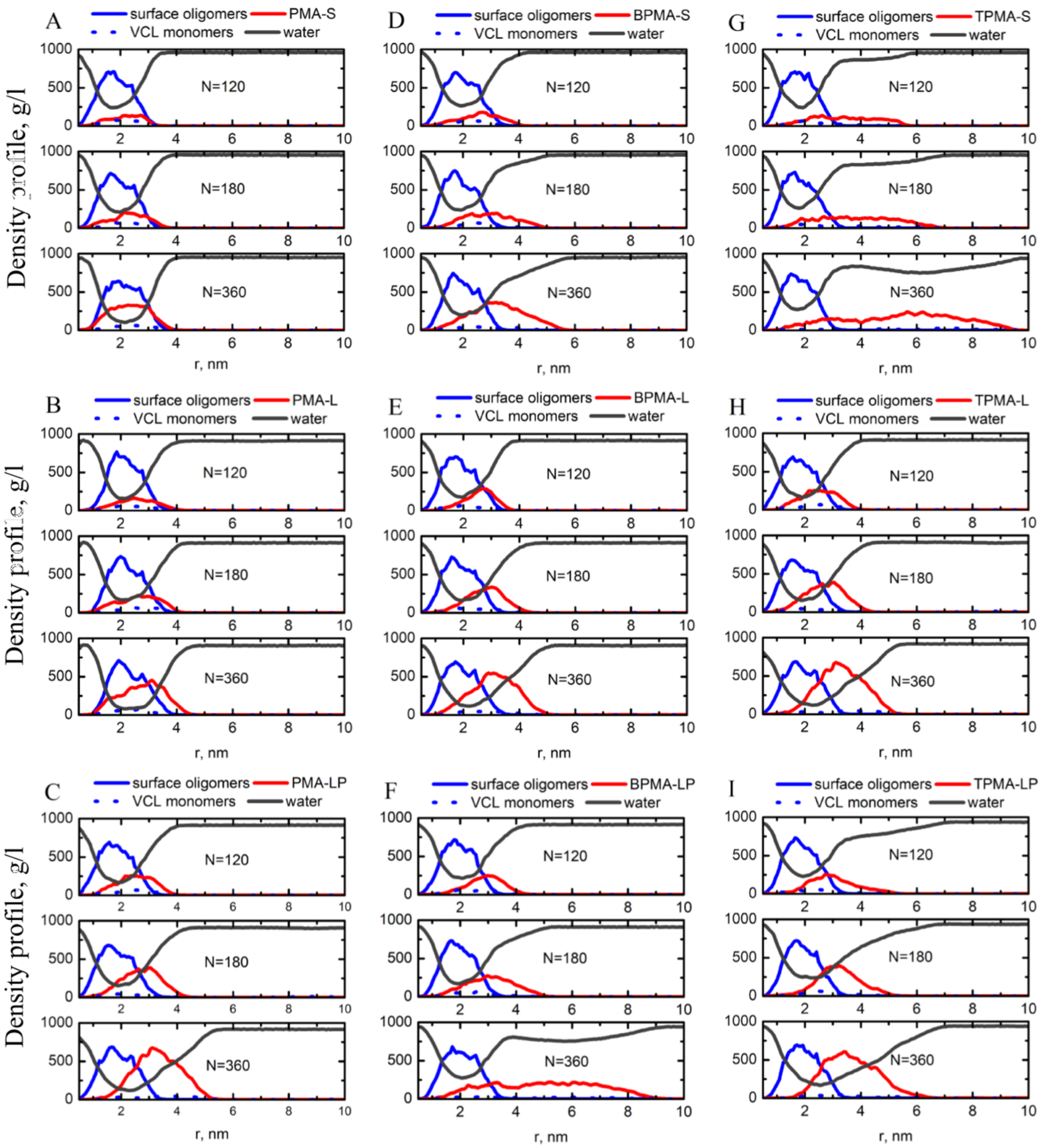

Figure S15. Density profiles for surface systems (A-I) along the normal, z-axis. The VCL oligomer and monomer distributions are shown in blue solid and dashed lines, respectively. Density profile of pyrazole residues is shown in red. Distribution of water molecules is depicted by a grey line.
